# Gene expression profiling describes the genetic regulation of *Meloidogyne arenaria* resistance in *Arachis hypogaea* and reveals a candidate gene for resistance

**DOI:** 10.1038/s41598-017-00971-6

**Published:** 2017-05-02

**Authors:** Josh Clevenger, Ye Chu, Larissa Arrais Guimaraes, Thiago Maia, David Bertioli, Soraya Leal-Bertioli, Patricia Timper, C. Corley Holbrook, Peggy Ozias-Akins

**Affiliations:** 1 Department of Horticulture and Institute of Plant Breeding, Genetics & Genomics, The University of Georgia, Tifton, GA 31793 USA; 2 Center for Applied Genetic Technologies and Institute of Plant Breeding, Genetics & Genomics, The University of Georgia, Athens, GA 30602 USA; 3 University of Brasília, Institute of Biological Sciences, Campus Darcy Ribeiro, 70910-900 Brasília, DF Brazil; 4 Embrapa Genetic Resources and Biotechnology, 70770-917 Brasília, DF Brazil; 5 USDA-ARS, Tifton, GA 31793 USA

## Abstract

Resistance to root-knot nematode was introgressed into cultivated peanut *Arachis hypogaea* from a wild peanut relative, *A*. *cardenasii* and previously mapped to chromosome A09. The highly resistant recombinant inbred RIL 46 and moderately resistant RIL 48 were selected from a population with cv. Gregory (susceptible) and Tifguard (resistant) as female and male parents, respectively. RNA-seq analysis was performed on these four genotypes using root tissue harvested from root-knot nematode infected plants at 0, 3, 7 days after inoculation. Differential gene expression analysis provides evidence that root-knot nematodes modulate biological pathways involved in plant hormone, defense, cell signaling, cytoskeleton and cell wall metabolism in a susceptible reaction. Corresponding to resistance reaction, an effector-induced-immune response mediated by an R-gene was identified in Tifguard. Mapping of the introgressed region indicated that 92% of linkage group A09 was of *A*. *cardenasii* origin in Tifguard. RIL46 and RIL 48 possessed 3.6% and 83.5% of the introgression on A09, respectively. Within the small introgressed region carried by RIL 46, a constitutively expressed TIR-NBS-LRR gene was identified as the candidate for nematode resistance. Potential defense responsive pathways include effector endocytosis through clathrin-coated vesicle trafficking, defense signaling through membrane lipid metabolism and mucilage production.

## Introduction

Root-knot nematodes (*Meloidogyne spp*.) are sedentary endoparasites affecting the root systems of over 3,000 plant species^1^. Among the three nematode species parasitizing peanut (*M*. *arenaria, M*. *hapla* and *M*. *javanica*), *M*. *arenaria* is the most prominent pathogen to the US peanut (*A*. *hypogaea*) industry due to its wide distribution in the peanut production regions^2^. The life cycle of *M*. *arenaria* starts from hatching in the soil as infectious second-stage juveniles (J2) which penetrate the root tip of a peanut plant. Inside the root, the J2 travels intercellularly along the vascular cylinder and remodels the parenchymatic cells into enlarged multinucleated giant cells as their feeding sites. Surrounding cells also exhibit hyperplasia and hypertrophy resulting in the formation of galls^3^. Within one month, a female nematode goes through three molts to become an adult and lays several hundred eggs outside the root. The growth of a nematode infested peanut plant can be severely stunted due to the disruption of root cell structure and shunting of nutrients to support nematode growth. Yield penalty is estimated at 3 to 15% in the infested peanut growing regions^4^.

Application of nematicides and soil fumigants are effective in reducing nematode populations, but are expensive to apply. Crop rotation between host and non-host crops can be effective, but host resistance is both effective and inexpensive for managing root-knot nematodes. Plant resistance genes *Mi* in tomato^5^, *Ma* in plum^6^, and *rhg1* in soybean^7^ confer strong resistance to nematodes. In *A*. *hypogaea*, only moderate host resistance to nematodes was identified among over one thousand plant introductions^8^. On the other hand, strong resistance to nematodes was revealed in a number of wild peanut relatives including *A*. *cardenasii* ^9, 10^. Introgression of nematode resistance into cultivated peanut was achieved through a three-way interspecific cross (*A*. *batizocoi* Krapov & Gregory) x (*A*. *cardenasii* x *A*. *diogoi* Hoehne) and intensive backcross programs^11–13^. Nematode resistant peanut cultivars COAN^12^ and NemaTAM^13^ were subsequently released. Tifguard^14^ was developed from a cross using COAN as the donor for nematode resistance. Tifguard carries a large alien introgressed region from *A*. *cardenasii* on chromosome A09 and inherits near immunity to *M*. *arenaria* ^15^. Recombination of the alien introgressed region was infrequent in the recombinant inbred population with susceptible peanut cultivar Gregory^16^ as female and Tifguard as male parent^15^. However, improved marker density on chromosome A09 allowed for the discovery of two rare recombinants, one carrying strong and the other moderate nematode resistance^17^. Utilizing these breeding lines to study nematode-host interaction provides an unique opportunity to dissect the resistant and susceptible responses.

Previously, suppression subtractive hybridization^18^ was employed to reveal differentially expressed genes between NemaTAM and a susceptible cultivar Florunner^19^. A total of seventy differentially regulated genes between the two cultivars were identified. A consititutive basal level of resistance in NemaTAM toward nematode challege was proposed. Profiling of host gene expression upon nematode challenge in other species has been performed with high-throughput arrays and RNA-seq analysis. Microarray analysis with nematode enriched root tissues from Arabidopsis^20^, tomato^21^ and laser capture microdissected (LCM) syncytia from soybean^22^ resulted in detection of thousands of differentially expression genes. RNA-seq analysis of LCM-isolated giant cells^23^ or root galls^24^ in rice and whole root tissue in alfalfa^25^ provided insights into the complex molecular pathways in response to nematode invasion including defense response, cellular transport, cell cycle, water channels, and cell wall metabolism. In this study, RNA-seq analysis was performed with nematode infected root tissues from Tifguard, Gregory, and two RILs demonstrating recombination within the alien introgressed region. Global gene regulation at early stages of nematode infection revealed molecular pathways conducive to nematode parasitism as well as cellular defense reactions leading to a resistance response. In addition, a candidate nematode resistance gene residing in the introgressed region is proposed.

## Materials and Methods

### Plant materials

The nematode resistant cultivar Tifguard, susceptible cultivar Gregory and two recombinant inbred lines, RILs 46 and 48 from a Gregory x Tifguard population were tested. RIL 46 and 48 exhibit strong and moderate resistance to root-knot nematode, respectively^17^. All plants were genotyped with two diagnostic KASP markers, i.e. Ah2n_TOG900848 and Ah2n_TOG898887^17^ for the alien introgressed region hosting strong and moderate resistance to nematode, respectively.

### Nematode inoculation and RNA extraction

Seeds were treated in a chlorine gas chamber for 8 hr prior to planting in sterilized vermiculite medium and watering with sterilized water^26^. Miracle-Gro (N-P-K 24-8-16) was applied two weeks after planting. Four weeks after planting, 20,000 J2 of *M*. *arenaria* worms were applied to each plant. Whole root tissue was collected with three biological replications for each treatment. For the day zero time point, only uninfected controls were collected. On three and seven days after inoculation (dai), both infected tissues and uninfected controls were collected. At the time of harvest, roots were washed with sterilized water and dried briefly with a paper towel before freezing and grinding in liquid nitrogen. Approximately 500 mg of the pulverized tissue was homogenized in RLT buffer using the Qiagen RNeasy Plant mini kit in proportions according to the manufacturer's instructions (Qiagen). Extracts equivalent to 80 mg of root tissue were used for RNA purification. Amplification grade DNAse I (Life Technologies) was used to treat RNA followed by concentration of treated RNA using the RNeasy MinElute Cleanup kit (Qiagen). Quality and quantity of RNA was determined by Agilent 2100 Bioanalyzer. RNA samples with RIN number greater than 8.0 were used for paired-end library construction with Kapa stranded RNA-seq kits (Kapa Bio Systems) at the Georgia Genomics Facility (Athens, Georgia).

### RNA sequencing and QC

Libraries were sequenced on an entire flow cell using the Illumina HiSeq 2500. De-multiplexed reads were checked for quality and kmer overrepresentation using FastQC (http://www.bioinformatics.babraham.ac.uk/projects/fastqc/). Reads were trimmed of 10 bases on the 5′ end due to kmer overrepresentation and to maintain high quality (median > 30) over the length of the read (100 bp). Trimmed reads were then mapped to a database of rRNA sequence to remove any rRNA contamination. Cleaned reads per library are shown in Table S1. Raw sequence for all samples is deposited at http://www.ncbi.nlm.nih.gov/bioproject/360316 at BioProject PRJNA360316.

### Introgression fine mapping

Reads were normalized to maximum coverage of 50 reads per kmer using Trinity normalization^27^ and mapped to the *A*. *duranensis* pseudomolecules^28^ (peanutbase.org) using Tophat v 2.1.0^29^ and the parameters ‘-I 5000 –no-mixed –no-discordant’. SNPs were called between Tifguard and Gregory using Samtools^30^ and SWEEP^31^. Custom scripts (supplied in Supplementary File S1) were used to identify SNPs between Tifguard and Gregory that also exist between *A*. *cardenasii* and *A*. *duranensis*. These SNPs were determined to be diagnostic for *A*. *cardenasii*. These SNPs were used to ‘trace’ the *A*. *cardenasii* introgression in Tifguard, RIL 46, and RIL 48.

### Read mapping and differential expression analysis

Cleaned reads were mapped to the *A*. *hypogaea* reference transcriptome assembly (PRJNA291488) using Bowtie^32^ and RSEM^33^. Percentage of reads mapped to the reference transcriptome ranged from 70% to 80%. Estimated counts were used for differential expression analysis using DESeq2^34^. Linear modeling differential expression analysis using three different comparisons and modeling seven different effects was carried out using the LRT() function. All p-values were corrected for multiple testing with a Benjamini-Hochberg correction with a false discovery rate (FDR) of 5%. Genes were determined to be differentially expressed with a corrected p-value < 0.05.

### Differentially expressed gene clustering using self-organizing maps

Groups of genes determined to be differentially expressed for each effect were clustered using the SOM (Self-Organizing Maps) function from the Kohonen Package in R^35^ using a 4 by 3 hexagonal SOM grid. Expression patterns of clustered genes were inspected manually for biologically relevant co-expression clusters.

### GO term enrichment analysis

GO terms associated with each cluster and total counts for each GO category in the full transcriptome assembly were counted using custom bash scripts. Counts for each sample (cluster) and population were used to calculate a p-value for enrichment using a hypergeometric test in R (phyper()). The resulting p-value for each represented GO term was adjusted by a Benjamini-Hochberg multiple testing correction. Finally, only p-values below the 0.001 level were considered, to further control for false positives.

### Tifguard, Gregory, and *Arachis cardenasii* whole genome shotgun sequencing

DNA from Tifguard, Gregory, and *Arachis cardenasii* (GKP10017; PI 262141) was extracted from seedling leaves using a Qiagen DNAeasy Plant mini kit® and sheared using a Covaris® sonicator to obtain 550 bp insert size. Sequencing libraries were constructed using the TruSeq Nano DNA Library Prep Kit (www.illumina.com). The libraries were quantified using Agilent DNA 7500 kit® on the Agilent 2100 Bioanalyzer. Sequencing was done on an Illumina MiSeq to generate 17,158,796 and 20,997,465 2 × 75 bp paired-end reads for Tifguard and Gregory, respectively, for roughly 1X coverage of the *A*. *hypogaea* genome. For *A*. *cardenasii*, a high-output run of the Illumina NextSeq was done at the Georgia Genomics Facility (Athens, GA; dna.uga.edu) generating 338,770,378 2 × 76 paired-end reads. Reads were checked for quality using FastQC^36^ and trimmed. Cleaned sequence was mapped to the *A*. *duranensis* v1 pseudomolecule^28^ (peanutbase.org) as the A genome reference sequence using BWA mem with default parameters^37^. Structural differences were identified by manual discovery of mapped reads where a single continuous read was split indicating an insertion/deletion.

## Results

### Fine mapping of *A*. *cardenasii* alien introgression on A09

**Figure 1:**
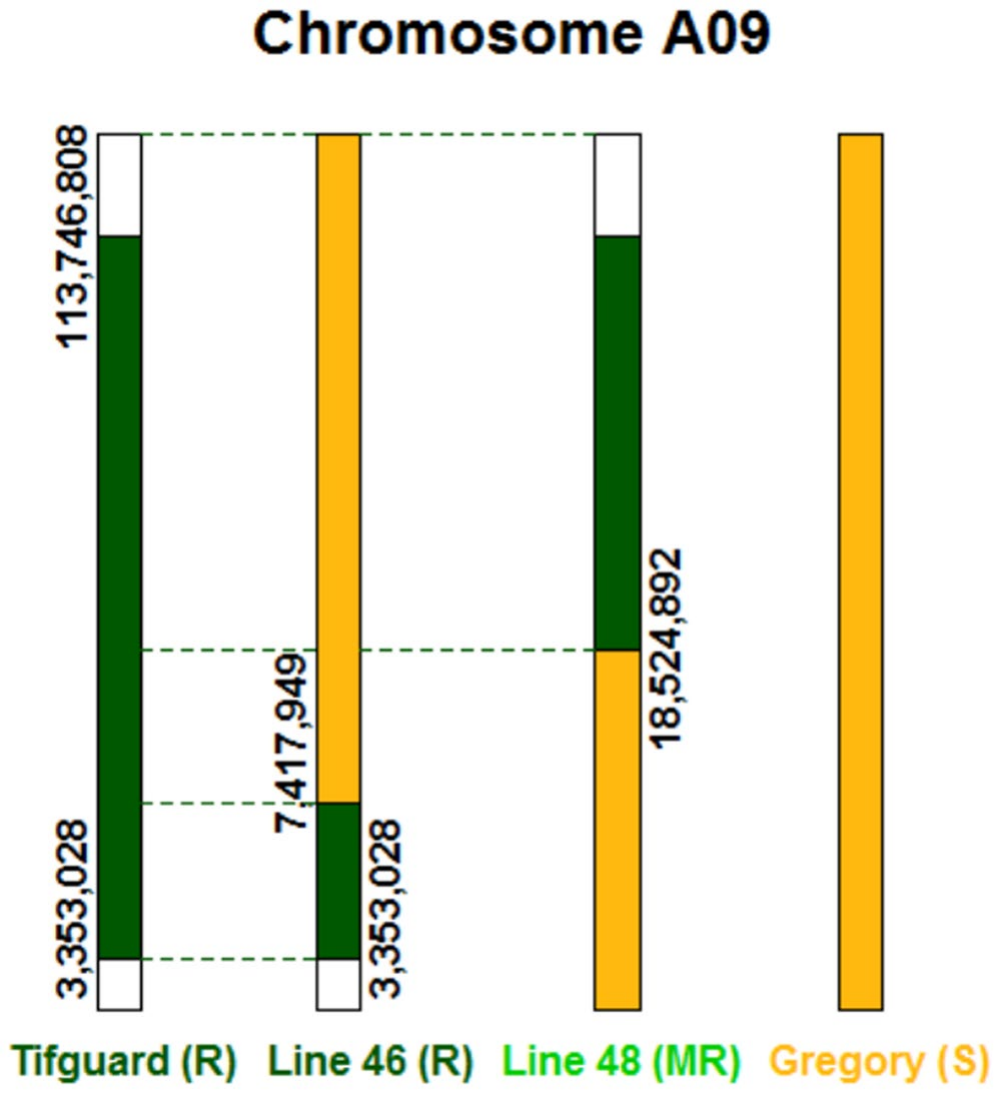
Physical map representation of *A*. *cardenasii* introgression in Tifguard, Line 46, and Line 48. Physical mapping is based on mapping of 555 SNPs diagnostic for *A*. *cardenasii* onto the *A*. *duranensis* reference pseudomolecule A09. Distinction between Gregory and Tifguard based on SWEEP filtered parental SNPs retained in each RIL line.

To map the introgression of *A*. *cardenasii* on chromosome A09 of *A*. *hypogaea*, a novel method was employed. First SNPs were called on chromosome A09 between Tifguard and Gregory and Tifrunner transcriptome using SWEEP^31^. Then SNPs were called between *A*. *cardenasii* and the *A. duranensis* genome as wild diploid diagnostic markers. Comparing the two sets of SNPs, any SNPs between *A*. *cardenasii* and *A*. *duranensis* were filtered out that were present in Gregory or Tifrunner (cultivated control). The SNPs remaining were a set of 555 SNPs describing the large A09 introgression in Tifguard ostensibly originating from *A*. *cardenasii*. The introgressed region on A09 spanned from nucleotides 3,353,028 to 113,746,808 which is 92% of the total length of chromosome A09. Figure 1 shows physical map coordinates of the introgression in Tifguard.

The same analysis was run on RILs 46 and 48 (Fig. 1). Each line contains a unique recombination in the introgressed region. RIL 46, which is nematode-resistant like Tifguard^17^, harbors a small region of the *A*. *cardenasii* introgression from 3,353,028 to 7,417,949 (Fig. 1). RIL 48 contains a much larger portion of the introgression, from 18,524,892 to 113,746,808 (Fig. 1). There is a region of the introgression absent from these two RIL lines, from 7,417,949 to 18,524,892. Based on phenotypic data^17^, the underlying molecular mechanism of the nematode resistance is conferred by the small introgression found in RIL 46. In one step the region has been fine mapped from 92% of the chromosome to 3.6% (4.06 Mb).

To confirm the *in silico* mapping, we developed and ran 14 KASP assays on Tifguard, Gregory, RIL 46, RIL 48, and controls NemaTAM, COAN, and Georgia-02C (susceptible). All of the 14 KASP assays corroborated the *in silico* mapping results (Figure S1; Table S2).

### Linear model analysis for differentially expressed gene groups

Instead of conducting pairwise differential expression analyses, a linear modeling-based analysis was adopted to identify genes that are differentially affected by genotype, treatment (nematode), and more importantly the interaction of genotype x treatment. To find these differentially expressed genes, reads were first mapped to the reference transcriptome assembly of *A*. *hypogaea*. This annotated set of mapped transcripts was assembled using the *A*. *duranensis* (A) and *A*. *ipaënsis* (B) genome sequence in order to differentiate between A and B homeologous gene pairs^28^.

Using the Likelihood Ratio Test (LRT) in DESeq2^34^, the effects of genotype, treatment, and genotype x treatment on differential expression of each gene were tested in three different line comparisons (Table 1).

**Table 1:** Number of differentially expressed genes for each comparison and percentage of differentially expressed genes compared to all expressed genes in the comparison.

Comparison	Effect	# of genes	% of expressed genes
Tifguard (R) vs Gregory (S)	Treatment	5,595	21.78%
	Genotype	5,371	20.91%
	Genotype x Treatment	1,739	6.77%
RIL 46 (R) vs Gregory (S)	Genotype	3,027	11.79%
	Genotype x Treatment	3,609	14.05%
RIL 48 (MR) vs Gregory (S)	Genotype	2,549	9.93%
	Genotype x Treatment	3,204	12.48%

Effects and interactions were tested using DESeq2 LRT test.

### The effect of nematode infection on gene expression

**Figure 2:**
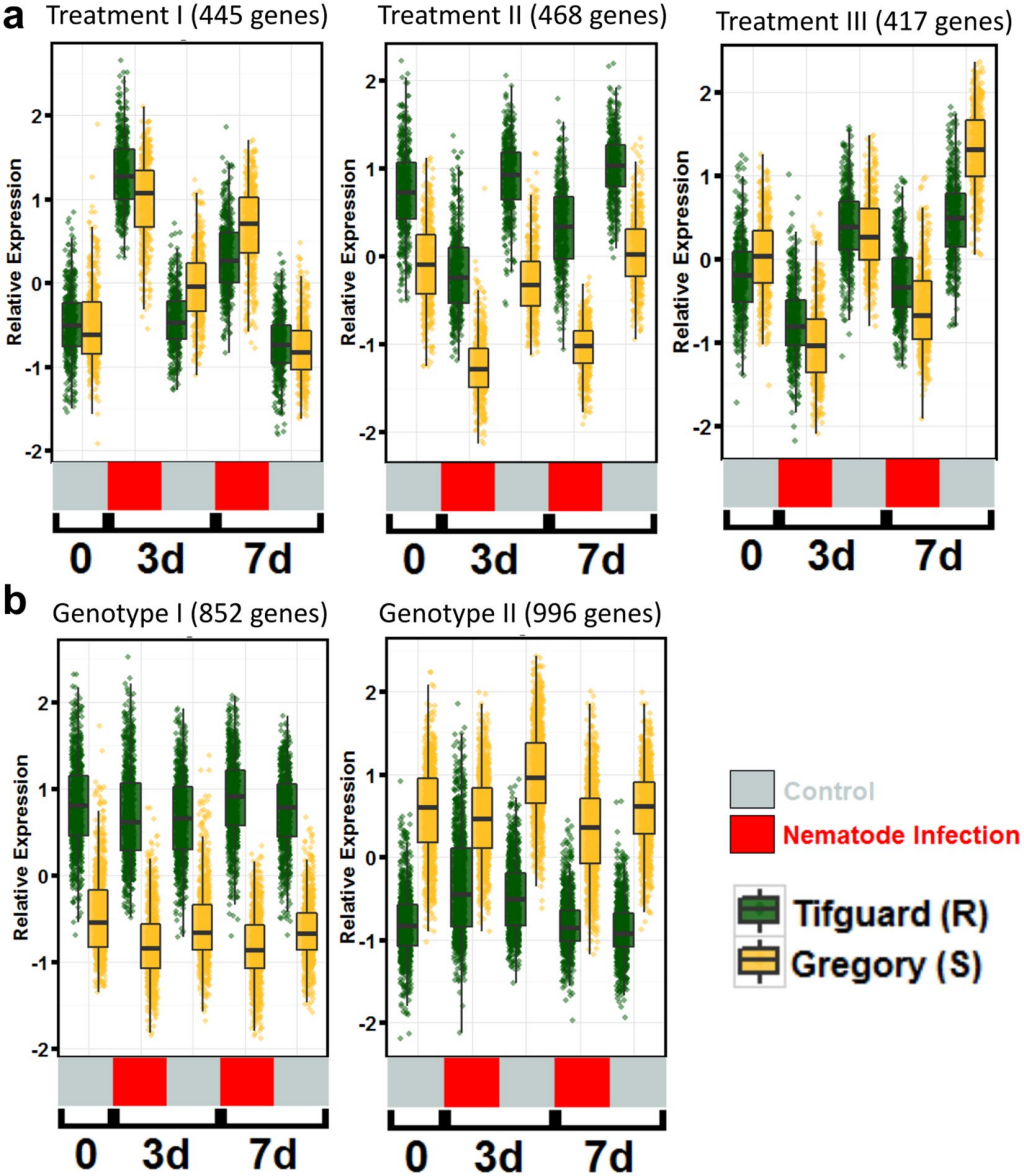
Co-expression clusters of significantly expressed genes affected by treatment and genotype. **(a)** Clusters representing genes affected by treatment (nematode infection). (**b)** Clusters representing genes affected by genotype. Jitter boxplots representing Z-score transformed relative expression of differentially expressed genes in each cluster. Boxplots represent median relative expression, first quartile (bottom of box), third quartile (top of box), and maxima. The actual spread of the data is represented by jittered dots so the relative expression of every individual gene is represented on the plot. Uninoculated control (silver) samples and samples inoculated with 20,000 juvenile *Meloidogyne arenaria* (red) and harvested at 3 days (3d) and 7 days (7d) after inoculation.

To differentiate the response to nematode infection from the actual resistance response, clusters of genes differentially expressed by nematode infection irrespective of genotype were examined. There were 5,595 genes differentially expressed in response to nematode infection. Three co-expression clusters (3,178 genes) were identified that describe the response to treatment (nematode infection) across genotypes, independent of resistance or susceptibility (Fig. 2a). The remaining 2,417 genes were excluded manually due to ambiguous expression patterns. Cluster I, nematode-responsive, shows genes that are up-regulated in response to nematode infection. GO term enrichment analysis revealed an up-regulation of genes related to the nucleolus, mRNA processing, and calcium-transporting ATPase activity.

The other two co-expression networks (clusters II and III) show a nematode-repressive response, i.e., down-regulation upon infection (Fig. 2a). Cluster II has 468 genes constitutively more highly expressed in Tifguard than Gregory, although both genotypes show the same repressive expression pattern. GO term analysis indicated a clear enrichment of microtubule-based movement, protein kinase activity, lignin catabolism, and ATPase activity (Table S3). Cluster III, composed of 417 genes, showed Tifguard and Gregory to have equal expression and down-regulation in response to nematode infection (Fig. 2a). GO term enrichment revealed response to nematode, polygalacturonase activity, response to osmotic stress, and transporter activity. These nematode responsive and repressive gene clusters will provide a backdrop against which to contrast the differential resistance and susceptibility response in Tifguard and Gregory, respectively (Table S3).

### Genes differentially expressed between genotypes independent of nematode infection

As described above, Tifguard harbors an alien introgression from *A*. *cardenasii* that covers 92% of chromosome A09 (Fig. 1). This large introgression will have effects on gene expression unrelated to any abiotic or biotic stresses. Introgressions have been shown to have effects on global gene expression in other crops^38^. It is possible that the resistance Tifguard confers is due to a constitutive expression difference as opposed to a differential response to the pathogen. A total of 5,371 genes are differentially expressed between Tifguard and Gregory by genotype effect, corresponding to 20.91% of expressed genes in the comparison (Table 1). Of these, 1,848 genes could be assigned to two clear groups where either the gene in Tifguard is constitutively more highly expressed (852 genes) or lower expressed (996 genes) compared to Gregory (Fig. 2b).

Top enriched GO terms for the set of genes constitutively up-regulated in Tifguard include aspects of cell proliferation encompassing mitosis, cell division, cell proliferation, microtubule-based movement, fatty acid beta oxidation, and DNA replication initiation (Table S4). These data suggest that the roots of Tifguard may have increased growth compared to Gregory. Mapping of these transcripts reveals an enrichment in localization to chromosome A09 by a chi-square test (p < 0.0001; Chi sq. 177.88; df = 9). Re-analysis of GO enrichment of the genes constitutively up-regulated in Tifguard that map to chromosome A09 reveal phosphatidylinositol-4,5 phosphatase activity, phosphatidylinositol phosphorylation, phosphatidylinositol-mediated signaling, oxylipin biosynthesis, and linoleate 13S-lipoxygenase activity (Table S4).

Top enriched GO terms for the set of genes constitutively down-regulated in Tifguard includes response to water deprivation, response to stress, protein dephosphorylation, protein serine/threonine phosphatase activity, and response to salt stress (Table S4). As above, there is an enrichment of genes constitutively down-regulated in Tifguard that map to chromosome A09 (p < 0.0001; Chi sq. 287.58; df = 9). Analysis of GO terms on these genes does not indicate any deviation from the larger cluster (Table S4). This cluster reveals that a suite of stress-related genes are constitutively down-regulated in Tifguard, again suggesting that the introgression has an effect on the gene expression of the roots that is not related to response to nematode infection. It cannot be ruled out that the resistance is due to a constitutive effect on gene expression.

### Genotype x treatment effect

**Figure 3:**
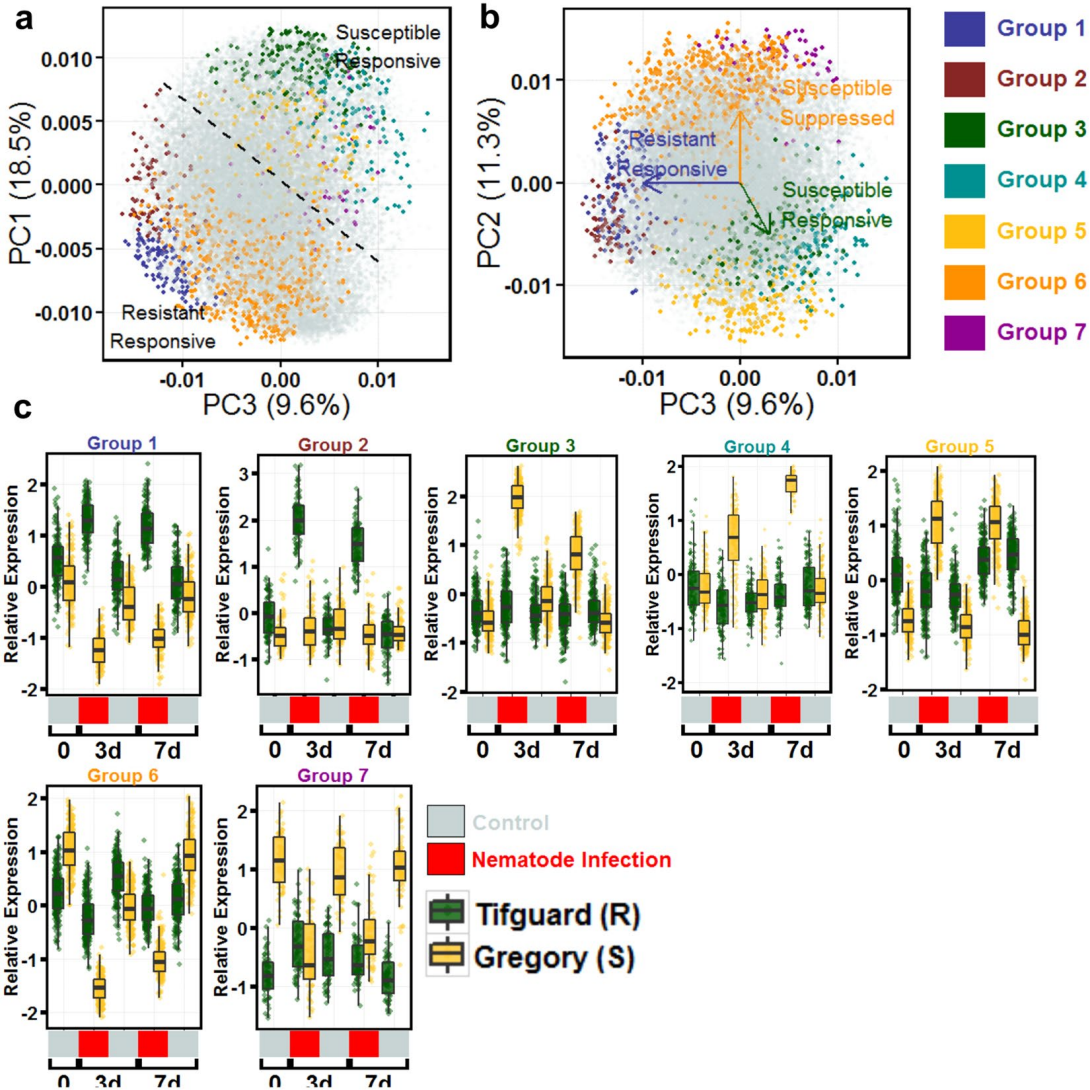
Genes differentially expressed affected by genotype x treatment. **(a)** PCA of all genes’ expression profiles with selected G x T gene co-expression networks highlighted. Groups can be separated into genes that respond to nematode treatment only in Tifguard (R) and those that respond only in Gregory (S). (**b)** Graphing PC2 and PC3 allows separation of the 7 co-expression groups into 3 categories, susceptible responsive, susceptible suppressed, and resistant responsive. (**c)** Boxplots of the expression patterns for each group. Boxplots represent median relative expression, first quartile (bottom of box), third quartile (top of box), and maxima. The actual spread of the data is represented by jittered dots so the relative expression of every individual gene is represented on the plot. Uninoculated control (silver) samples and samples inoculated with 20,000 juvenile *Meloidogyne arenaria* (red) and harvested at 3 days (3d) or 7 days (7d) after inoculation. Group I (141 genes); Group 2 (81 genes); Group 3 (197 genes); Group 4 (132 genes); Group 5 (184 genes); Group 6 (653 genes); Group 7 (62 genes).

**Figure 4:**
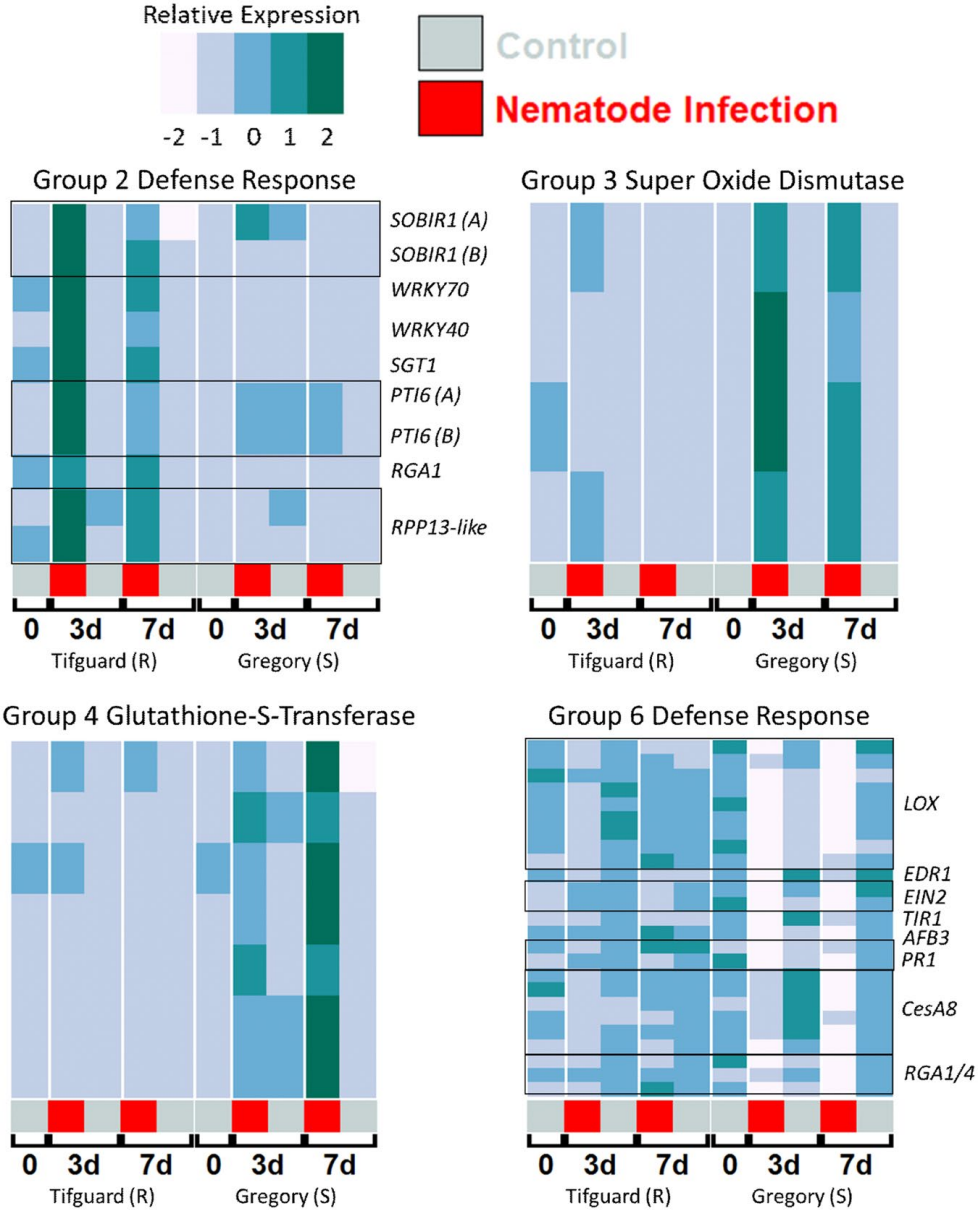
Expression heatmaps of individual differentially expressed genes from top GO enriched categories of selected groups. Individual genes representing top enriched GO terms from genotype x treatment-affected, differentially expressed gene clusters Group 2 (Resistant Responsive; “Defense response”), Group 3 (Susceptible Responsive; “Super Oxide Dismutase Activity”), Group 4 (Susceptible Responsive; “Glutathione Transferase Activity”), and Group 6 (Susceptible Suppressed; “Defense Response”). Heatmap expression is Z-score normalized relative expression. Uninoculated control (silver) samples and samples inoculated with 20,000 juvenile *Meloidogyne arenaria* (red) and harvested at 3 days (3d) or 7 days (7d) after inoculation. Black boxes in the heatmap indicate the genes are from the same family. For *PTI* and *SOBIR1* the two genes are putative A and B homeologous copies.

There were a total of 1,739 genes differentially affected by the interaction of genotype and treatment (nematode). To differentiate the interaction responses, genes were further classified using self-organizing maps. Seven co-expression networks of interest were identified with expression patterns that distinguish the resistance from susceptible reaction to nematode treatment (Fig. 3a). Principle components analysis (PCA) showed that the groups can be separated into three categories – resistance responsive (Groups 1 and 2), susceptible responsive (Groups 3, 4, and 5), and susceptible suppressed (Groups 6, and 7) (Fig. 3b). Groups 1 (81 genes) and 2 (140 genes) (Fig. 3c) show genes that are up-regulated in response to nematode treatment in Tifguard but not in Gregory. Group 1 additionally shows that in Gregory, these genes are down-regulated in response to nematode treatment. Enriched GO terms for Group 1 shows a defense response, with the top enriched terms being ubiquitin ligase complex, positive regulation of cell death, kinase activity, and cyclic nucleotide channel activity (Table S4). Enriched GO terms for Group 2 include defense response (Fig. 4) and cyclic nucleotide channel activity, but also include three classes of acylhydrolase activity, galactolipase, phosphatidylcholine-1-acylhydrolase, and triglyceride (TAG) lipase activity (Table S5). Acylhydrolases have been shown to be involved in defense-mediated jasmonic acid biosynthesis^39^. These groups of differentially regulated genes up-regulated in Tifguard provide evidence that the resistance may be mediated by an R-gene incompatible reaction.

Groups 3 (197 genes), 4 (132 genes), and 5 (184 genes) show genes up regulated in susceptible Gregory in response to nematode treatment that are unresponsive in resistant Tifguard (Fig. 3c). Group 3 shows an early response and enriched GO terms superoxide dismutase activity (Fig. 4), glutamate metabolic process, ATP hydrolysis coupled proton transport, and regulation of DNA replication (Table S5). Group 4 shows a late response, being up regulated at 7 dai (Fig. 3c). Top enriched GO terms for Group 4 include spermine, and putrescine biosynthesis, cysteine biosynthesis activity, and glutathione transferase activity (Fig. 4; Table S5). Group 5 is interesting because in Gregory the genes exhibit very low expression but are up regulated in response to nematode infection similarly at 3 and 7 dai. Top enriched GO terms for Group 5 include ribosome, translation, DNA-directed RNA polymerase, electron transport, and glycolysis (Table S5). In resistant Tifguard, the genes in Group 5 are more highly expressed than in Gregory but are not responsive to nematode treatment (Fig. 3b).

Groups 6 (653 genes) and 7 (62 genes) are down regulated in response to nematode treatment in susceptible Gregory but not in resistant Tifguard (Fig. 3). GO terms include secondary cell wall biogenesis, linoleate lipoxygenase activity and oxylipin biosynthesis, defense response, indole-3-acetic acid amino synthetase activity, cellulose synthase, and DNA binding/transcription factor (Fig. 4). Enriched GO terms contain many components of defense response (Table S5). Group 7 genes provide an additional element. Their locations are enriched on chromosome A09 (p < 0.0001; Chi sq. = 97.36; df = 19). Top enriched GO terms for Group 7 include auxin homeostasis, response to auxin, and camalexin biosynthesis (Table S5).

### RIL 46 genotype x treatment effect

If the data are analyzed using RIL 46 as the resistant genotype, the response to nematode invasion can only be delineated relative to the major resistance located within the 4 Mb alien introgression. The phenotypic outcome of RIL 46 is the same as Tifguard, near immunity, but the genetic response is slightly different because RIL 46 only contains three percent of the *A*. *cardenasii* introgression (Fig. 1). There were 3,609 genes determined to be differentially expressed by genotype x treatment between RIL 46 and Gregory (Table 1). Of those, 818 genes are in common with Tifguard. Comparison of estimates of log_2_ (fold change) between Tifguard and Gregory and RIL 46 and Gregory shows strong correlation between Tifguard and RIL 46, indicating that this genetic response can be attributed to the shared 4 Mb *A*. *cardenasii* introgression on chromosome A09 (Figure S2; R-squared = 0.91; p < 0.001). Clustering those 818 genes reveals three main groups of interest (Figure S3). Group I (143 genes) consists of genes that are upregulated in response to nematode treatment (especially at 7 dai) in RIL 46 that are down regulated in Gregory (Figure S3A). Top enriched GO terms for Group I include ADP and ATP binding, defense response, signal transduction, and protein serine/threonine protein kinase activity, providing evidence that the genetic response of 46 and Tifguard to nematode treatment that differentiates them from susceptible Gregory is an R gene-mediated defense response (Table S6).

### RIL 48 genotype effect

RIL 48 exhibits a moderate, quantitative resistance phenotype in response to *M*. *arenaria*, and carries a large region of the *A cardenasii* introgression that is non-overlapping with the 4 Mb region in RIL 46 (Fig. 1). RIL 48 presents an opportunity to explore the possible moderate resistance QTL^40^ and contrast the genetic response with the major resistance in RIL 46.

The genotype comparison between RIL 48 and susceptible Gregory yields 3,027 differentially expressed genes (Table 1). Of those, 1,027 are in common with the Tifguard comparison with Gregory. If compared to the RIL 46 comparison with Gregory, of the 1,027 genes, 628 are uniquely differentially expressed between RIL 48 and Tifguard. These genes potentially tell the story of the moderate resistance. Analysis with the Tifguard comparison with genotype shows that the fold change estimates are correlated (Figure S4; R-squared = 0.591; p < 0.001). The moderate resistance in RIL 48 is potentially conferred by a constitutive difference in expression compared with Gregory since no genes were significantly upregulated in response to nematode treatment in RIL 48 compared with Gregory in the genotype x treatment interaction analysis (data not shown). This difference allows RIL 48 to avoid severe consequences of nematode infection while lacking a strong incompatible reaction like RIL 46 and Tifguard. Further, this constitutive difference will be shared with Tifguard because it is controlled genetically by the 95 Mb alien introgression that Tifguard and RIL 48 share.

**Figure 5:**
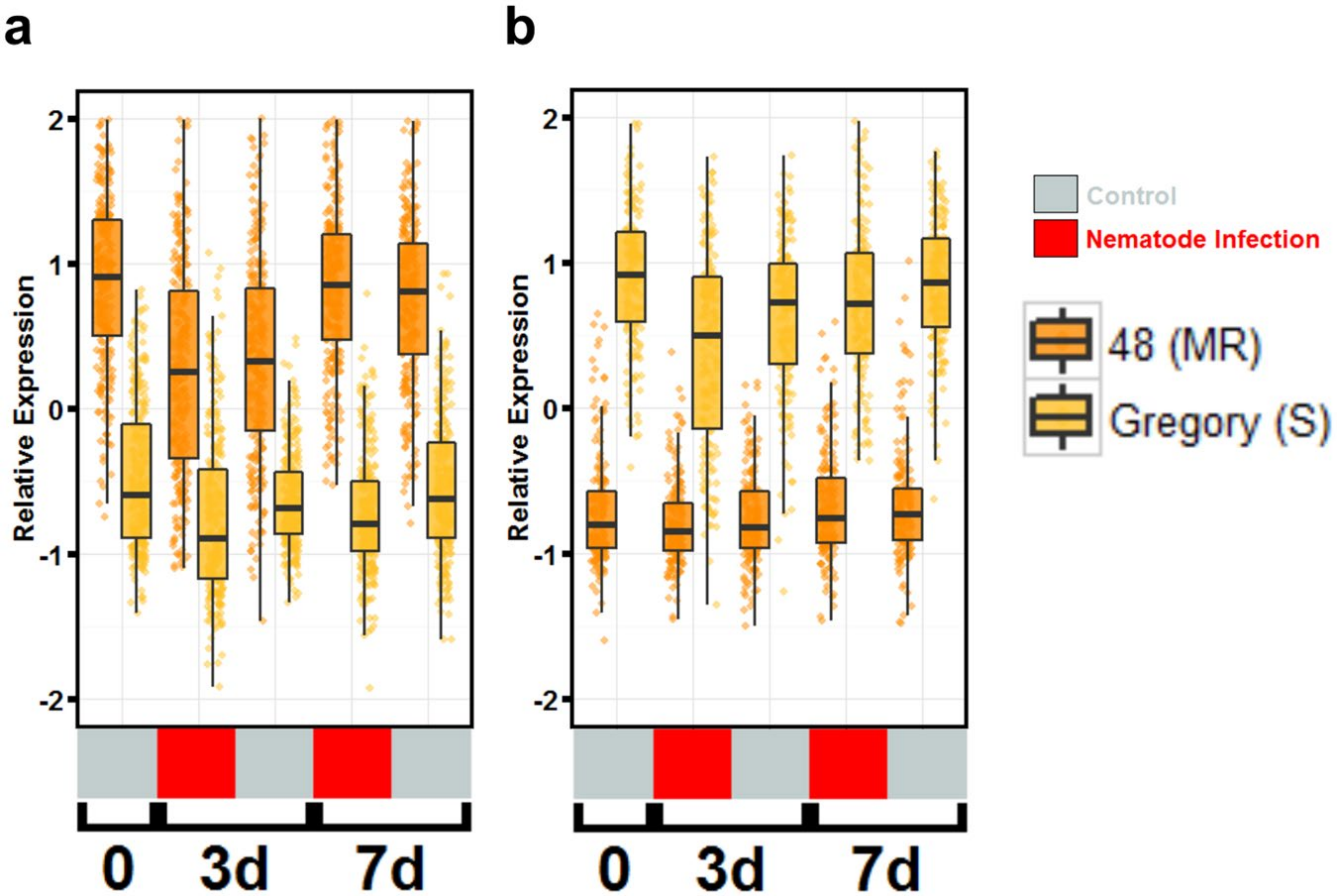
Differentially expressed genes affected by genotype between RIL 48 (MR) and Gregory (S). (**a**) Genes constitutively more highly expressed in line 48 compared to Gregory and (**b**) genes constitutively lower expressed in line 48 compared to Gregory. Boxplots represent median relative expression, first quartile (bottom of box), third quartile (top of box), and maxima. The actual spread of the data is represented by jittered dots so the relative expression of every individual gene is represented on the plot. Uninoculated control (silver) samples and samples inoculated with 20,000 juvenile *Meloidogyne arenaria* (red) and harvested at 3 days (3d) or 7 days (7d) after inoculation.

The 628 genes have two dominant expression patterns, either constitutively more highly expressed in RIL 48 or constitutively more highly expressed in Gregory (Fig. 5). There are 256 genes constitutively up regulated in RIL 48 (Fig. 5a). Of the 256 genes, 38% (96 genes) map to chromosome A09 (38) or B09 (58) which is a significant enrichment based on the chi-square test (p < 0.001; chi sq. = 248.229; df = 19). Of those genes mapping to chromosome A09, none maps to the 4 Mb region of the alien introgression retained in RIL 46. This combined with the observation that these genes are not differentially expressed in RIL 46 compared to Gregory suggests that the differential expression of these genes seen in RIL 48 can be attributed to the *A*. *cardenasii* introgression this line retains. Top enriched GO terms for this group of genes shows enrichment of phosphatidylinositol-mediated signaling with GO terms phosphatidylinositol dephosphorylation, and phosphatidylinositol-3,4,5-trisphosphate 3-phosphatase activity (Table S7). The phosphatidylinositol pathway has been shown to be important for defense response^41, 42^, and mutants in this pathway can compromise hypersensitive response-mediated defense and have a weaker basal defense response to elicitors^43, 44^.

The other set of 175 genes are constitutively over-expressed in Gregory relative to Line 48 (Fig. 5b). Of the 175 genes, 89 (51%) map to chromosome A09 which is also highly significantly enriched by a chi-square test (p < 0.0001; chi sq. = 793.8; df = 19). Again, not one of these genes maps within the 4 Mb introgression present in RIL 46. Top enriched GO terms for this group include oxylipin biosynthesis, linoleate 13S-lipoxygenase activity, and lipid modification. Interestingly, there are genes within this group that are important for defense response, including a putative ortholog of *ENHANCED DISEASE RESISTANCE1* (EDR1) which negatively regulates defense response, a putative ortholog of WRKY40, and a putative TMV resistance protein N.

### Novel genes in Tifguard and identification of a candidate gene for resistance

Tifguard contains an introgression from *A*. *cardenasii*, therefore it is possible that there are novel expressed transcripts in Tifguard. To investigate this, a *de novo* assembly strategy was used to assemble all Tifguard-specific transcripts, and the assembly was tested to ensure that no reads from Gregory mapped to these transcripts. Finally, only putative novel transcripts from Tifguard that mapped to chromosome A09 were considered, of which 63 were identified. Of the 63, only seven mapped to the region shown to contain the high level of resistance and three were annotated as disease resistance genes. Only one of the three had no hit in the *A*. *hypogaea* reference transcript set (ncbi.nlm.nih.gov; PRJNA291488). Although the other two cannot be ruled out as candidates, they are present in *A*. *hypogaea* transcriptome assemblies and so are less likely candidates. This candidate transcript is not highly expressed in Tifguard, but is constitutively expressed at a low level. Using RT-PCR, we confirmed that this gene was expressed in Tifguard and RIL 46, but not in Gregory or RIL 48 (Figure S5), validating the RNA-seq results. Furthermore, this gene was not expressed in 22 different tissues of *A*. *hypogaea* cv. Tifrunner, which is also susceptible to nematodes (peanutbase.org; Figure S6), suggesting it to be a candidate gene for resistance.

To further investigate this candidate resistance gene, shotgun re-sequencing of Tifguard and Gregory was carried out. Using the diploid *A*. *duranensis* chromosome A09 pseudomolecule as a reference, two insertions/deletions (indels) were discovered between the *A*. *cardenasii* lineage in Tifguard and the *A*. *duranensis* lineage in Gregory for the candidate R gene along with 34 exonic SNPs (Figure S7). The first intron of Gregory contains a retrotransposon insertion that could be affecting transcription. Further, there is a 94 bp indel in exon 4, which corresponds to the LRR domain. Both of these structural variations were confirmed by PCR and Sanger sequencing (Figure S8). No structural variation in the promoter region of the candidate gene was detected, although SNPs are present. This candidate gene will be investigated further for its role in nematode resistance in *A*. *hypogaea*.

## Discussion

### Gene regulation in compatible reactions

Extensive host plant gene regulation has been reported upon nematode infection^45^. In a compatible reaction, root-knot nematodes evade and suppress the host native immune response mediated by the recognition of pathogen-associated-molecular patterns (PAMPs) and initiate feeding sites to shunt plant metabolites for support of growth and reproduction. Well-developed giant cells associated with swollen J2 nematodes were observed seven days after inoculation (DAI) in susceptible peanuts^10, 46^. In an incompatible reaction, similar numbers of J2s were observed in the roots of *A*. *cardenasii* at 7 DAI yet no giant cells were formed, none of the J2s were able to develop further, and a hypersensitive reaction characterized by host cell necrosis surrounding J2s was reported^10^. TxAG-6^11^, the tetraploid interspecific predecessor of Tifguard, inherited the near immunity to root-knot nematode from *A*. *cardenasii*, yet no host cell necrosis was observed at 56 days post inoculation^46^.

Since the *M*. *arenaria* J2s are able to invade roots of both resistant and susceptible peanut cultivars, common host responses between the two types plus unique response of a susceptible genotype to nematode infection characterize the compatible reactions to nematode invasion. A total of 2,568 genes responsive to nematode infection as a consequence of compatible reaction were clustered in the current study (Figs 2a and 3c groups 3–7). GO terms related to compatible reaction across the clusters were compiled and a subset with a clear function category is discussed below (Table S8).

In the plant hormone category, genes involved in auxin homeostasis and auxin signaling pathway are mostly down regulated in both Tifguard and Gregory except for a subset of genes in the auxin homeostasis pathway which was up-regulated in Gregory and had no change in Tifguard. Nematodes have been known to hijack the host plant hormonal signaling pathways to establish a feeding site^45, 47^. Auxin regulates plant cell cycle and organogenesis and is responsive to developmental and external cues^47^. Increased auxin transport and accumulation at the feeding site of root-knot nematode was reported in white clover^48^. Nematode infection was found to alter expression of *PIN* auxin transporters resulting in the redistribution of auxin to the cells surrounding feeding sites^47^. Differentially regulated auxin-related genes were reported in the root tips of nematode infected tomato^21^. Abscisic acid (ABA) synthesis was down regulated whereas ABA transport was up-regulated in Gregory. Jasmonic acid (JA) and ethylene (ET) mediated signaling pathways were down regulated in Gregory. JA and ET are phytohormones known to be the key players in plant defense against pathogens^49^. JA and ET signaling pathways are responsive to mechanical wounding and herbivore predation. ABA has been shown to counteract the defense signals regulated by JA and ET and promote host susceptibility to pathogens^50, 51^. Suppression of JA/ET dependent pathway associated genes were identified in root-knot nematode infected Arabidopsis^20^. Therefore down regulation of JA/ET signaling pathways in Gregory possibly promotes nematode susceptibility.

In the defense category, biological pathways regulated similarly between Gregory and Tifguard include isoflavonoids, ceramide metabolic pathways and general defense response. Isoflavonoid phytoalexins had been shown to be part of host defense against nematodes in alfalfa^52^ but did not play a role in feeding site formation^53^. Ceramide, a bio-reactive sphingolipid, is extensively involved in programed cell death^54^. In this experiment, both ceramidase and ceramide catabolic process were down regulated which possibly resulted in accumulation of ceramide. Environmental stress has been shown to increase plant ceramide levels^55^. Common response between the resistant and susceptible cultivars suggests that these defense components could be regulated by mechanical wounding caused by the migration of nematodes through the roots. Camelexin biosynthesis was down regulated only in Gregory and showed no change in Tifguard upon nematode challenge. Camalexin is a well-known antimicrobial compound. An Arabidopsis mutant line with impaired camalexin synthesis supported significantly more gall production than the wild type^56^. In the cell signaling category, phosphoinositide synthesis and degradation were modulated similarly between Gregory and Tifguard upon nematode invasion. It has been shown that phosphoinositides are critical plant defense components participating in salicylic acid (SA) mediated defense^41, 42^. Stress can induced dynamic changes of phosphoinositide levels and endomembrane rearrangement^57^. On the other hand, up-regulation of glutamate metabolism and down regulation of oxylipin biosynthesis and linoleate 13S-lipoxygenase activity were specific to Gregory. Glutamate metabolism has been shown to be exploited by pathogens to facilitate infection^58^. Oxylipins are oxidative derivatives of unsaturated fatty acids that often elevate in production upon pathogen challenge^59^. They act as signal molecules or provide protective compounds regulated by the JA signaling pathway. Suppression of JA signaling and down regulation of oxylipin synthesis facilitated nematode susceptibility in Gregory.

Root-knot nematode infection causes significant host gene regulation related to cell wall modification and cytoskeleton responses in susceptible plants. At the early stages of infection, *M*. *arenaria* alters the cell cycle of vascular parenchyma cells with multiple mitoses skipping cytokinesis resulting in giant cells with more than 100 nuclei^60, 61^. Surrounding cells of the enlarged giant cells proliferate to form galls^60, 61^. Cell wall ingrowth near xylem and cytoskeleton fragmentation are involved in the formation of these feeding cells of nematodes^60, 61^. In both peanut genotypes, cell wall modification involved in abscission, cellulose synthesis, lignin and pectin catabolic processes were down regulated whereas endo-β-D-glucanase (EGase) activity was up regulated. Cell wall pectin synthesis was up regulated in Gregory specifically. Cellulose forms the cross-linked microfibril which is the main component of primary plant cell walls. Pectin matrix is embedded in the cell wall microfibril network providing wall porosity. Lignins are cross-linked phenolic polymers functioning as the main components of secondary cell walls^62^. Nematodes are known to regulate cell wall biosynthesis and degradation pathways to achieve extensive cell wall architectural modification^60^. EGases hydrolyze the outermost layer of non-crystalline cellulose of the cell wall. Up-regulated EGase expression was reported in the nematode-infected susceptible peanut cultivar Florunner^18^ and tobacco suggesting its roles in loosening the cell wall for the formation of nematode feeding sites^60, 63^. Pectate lyases and the cellulose synthase gene family previously were shown to be up regulated in either giant cells or gall tissues^64^. However in our study, both pectate lyase and cellulose synthase were down regulated. The discrepancy may have been caused by differences in tissue types under study. Whole root tissue was sampled in this study whereas giant cells or gall tissues were sampled in the previous study^64^. As for biological pathways involved in cytoskeleton metabolism, actin filament assembly and microtubule complex formation were down regulated in both genotypes. Random and diffuse actin filaments and microtubules were reported in giant cells formed by nematodes^61^. Stabilization of the microtubular cytoskeleton prevented feeding site formation whereas elevated expression of actin depolymerizing factors (ADF) promoted it^61, 65^. Therefore, the down regulation of actin- and microtubule-associated pathways detected in this study may be associated with the initiation of feeding sites. Since both resistant and susceptible genotypes responded similarly in these pathways, the most critical biological process sustaining successful feeding site formation lies beyond these pathways under discussion.

### Gene regulation in the incompatible reaction

A set of defense responsive genes in the categories of plant hormones, defense and cell signaling were up regulated in Tifguard and were either down regulated or unchanged in Gregory (Table S9 incompatible). Enhanced negative regulation of ABA biosynthetic process in Tifguard suggests lower accumulation of ABA. It was previously shown that application of ABA antagonized the basal defense system signaled by SA/JA/ET and increased susceptibility to migratory nematodes in rice^66^. Suppression of ABA synthesis in Tifguard possibly plays a role in preventing the establishment of feeding sites. In the defense category, biological pathways involved in defense responses, clathrin-coated vesicles, protein ubiquitination, mucilage, tyrosine and serine/threonine kinase metabolism were up regulated. Clathrin-coated vesicles form at plasma membranes upon recognition and packaging of specific cargoes for endocytosis^67^. This process is known to be involved in plant defense against bacteria and fungi^68^. As a part of pathogen-associated molecular pattern (PAMP)-mediated defense, ubiquitination of bacteria flagillin receptor FLS2 through ubiquitin ligase provided signals for toxin endocytosis and degradation^69^. Up-regulation of both ubiquitination and clathrin-coated vesicle assembly could be implicated in the defense response of Tifguard, suggesting that nematode effector proteins are sequestered in Tifguard through this clathrin-coated vesicle pathway. Both tyrosine and serine-threonine kinases are involved in plant development and disease resistance. They participate in the signal relay of PAMP-regulated plant defense through mitogen-activated protein kinase (MAPK) cascades^70, 71^. Mucilage can be produced by root border cells and presented as a polysaccharide layer^72^. Protective effects of mucilage layer against bacterial infection^73^ was reported previously. Overproduction of mucilage inside the root xylem causes blockage of parasite supply channels; this was identified as the defense mechanism against broomrape in vetch (*Vicia* spp.)^74^. Increased mucilage metabolic pathways in Tifguard could contribute to the prevention of nematode feeding site formation. Positive regulation of several pathways related to defense response and cell death suggest that a hypersensitive response could be involved in the incompatible reaction.

In the cell signaling category, up regulation of phosphatidylcholine metabolism, phosphoinositol binding and phospholipase D activity in Tifguard suggests the strong resistance to nematode is possibly orchestrated through the dynamic membrane lipid metabolism and signaling. Phosphatidylinositol bisphosphate (PIP2) regulates phospholipase D (PLD) hydrolysis of phosphatidylcholine, phosphatidylethanolamine and phosphatidylglycerol to produce stress responsive second messenger phosphatidic acid (PA)^75^. PLD and PA have wide implications in hormonal signaling and plant defense response against pathogen invasion^76^. Subsequent modification of cytoskeleton and microtubule structure by PLD and PA may facilitate clathrin-coated vesicle trafficking for pathogen elicitor degradation. Cyclic nucleotides were found to regulate plant defense response downstream of nitric oxide^77, 78^. Cyclic nucleotide-gated ion channels are the receptors of cyclic nucleotides that modulate Ca^2+^ influxes in response to pathogen invasion. Up regulation of both cyclic nucleotide binding and cyclic nucleotide-gated cation channel activity in Tifguard implies a function in nematode resistance.

### Comparison of responsive pathways from RIL 46 and 48

The recombinant lines RIL 46 and RIL 48 carry 3.6% or 83.5% of chromosome A09 introgressed from *A*. *cardenasii*. It is therefore expected that the expression profile of RIL46 and RIL 48 would share similarity to Tifguard. Comparing the constitutively more highly expressed genes in RIL 48 and Tifguard using Gregory as a check, 57 out of 136 GO terms (Figs 2b and 5a left panel) co-occurred and 89% of them mapped to chromosome A09. A few members of the phospholipid signaling pathway are in this group suggesting their potential defense role^41^ to maintain the intermediate resistance encoded by the introgressed region. As for the constitutively lower expressed genes compared to Gregory, 13 out of 86 GO terms (Figs 2b and 5b right panel) were in common between RIL 48 and Tifguard. Seven GO terms (54%) were mapped to chromosome A09. Decreased mitochondrial transport and glucose metabolic process in this cluster could be the consequence of intermediate resistance response directed by chromosome A09.

### Discovery of a candidate resistance gene

Cultivated peanut (*A*. *hypogaea*) is an allotetraploid with two sets of homeologous subgenomes AABB. Two wild diploid species *A*. *duranensis* (A genome) and *A*. *ipaënsis* (B genome) were the progenitors and gave rise to allotetraploid peanut through interspecific hybridization and natural polyploidization^78^. Both subgenomes share greater than 98% sequence similarity to cultivated peanut and their genome sequences are publically available^28^ (peanutbase.org). The introgressed nematode resistance region in Tifguard came from another A-genome diploid species *A*. *cardenasii*. Since the cultivated peanut genome sequence is not available, SNP calling between *A*. *cardenasii* and *A*. *duranensis* on chromosome A09 provided diagnostic markers for the *A*. *cardenasii* introgression. Further subtraction of common SNPs shared with susceptible peanut cultivars allowed for accurate delineation of the introgressed region in Tifguard which spans 92% of the A09 chromosome (Fig. 1). Strongly resistant RIL 46 possesses only 4.06 Mb (3.6%) of the introgressed region suggesting that dominant gene(s) conditioning nematode resistance reside in this small introgressed region. RIL 48 has 83.5% of the introgressed region and exhibits moderate resistance suggesting that this region may condition nematode resistance quantitatively. This method of comparative physical mapping of differentially expressed genes is highly useful for traits demonstrating severely suppressed recombination.

*De novo* assembly of Tifguard specific transcripts led to the discovery of a candidate resistance gene residing in the small introgressed region of RIL 46. The Toll/Interleukin 1 Receptor (TIR)-Nucleotide Binding Site (NBS)-Leucine Rich-Repeat (LRR) domain is characteristic of an R-gene mediating immune response in plant-pathogen interactions^79^. The C-terminal LRR domain involved in protein-protein interaction which could potentially recognize effector proteins released by the nematode pathogen. The presence of a NBS domain suggests that the candidate R gene activity may require ATP binding or hydrolysis^80^. The N terminal TIR domain could initiate downstream signaling for defense responses^81^. There are two classes of plant R genes possessing the NBS-LRR domain. The first class encodes the N-terminal TIR-NBS-LRR domain such as the candidate gene of our interest and previously identified nematode resistance genes *Gro1-4* in potato^82^ and *M*a in plum^6^. Our candidate R gene shared 31% sequence similarity with these previously published resistance genes (Figure S9). Both TIR and kinase domains are highly conserved whereas the LRR region is more variable in the candidate R gene compared to the published resistance genes. The second class has a N-terminal coil-coil (CC) –NBS-LRR domain such as nematode resistance genes *Mi-1*.*2, Mi-9*, and *Hero* in tomato^5, 83, 84^. It has been proposed that binding of pathogen effectors to the LLR domain promotes the exchange of ADP to ATP binding of the NBS domain and subsequent signal transduction for defense response^85^. The lack of expression of this candidate gene in susceptible peanut cultivar Gregory (Figure S5) and moderately resistant line RIL 48 (Figure S5) and its constitutive expression in resistant lines Tifguard and RIL 46 (Figure S5) warrants further functional investigation. Genome editing of the candidate gene is currently in progress.

From R-gene expression and stress responsive genes in Tifguard, a model of the resistance mechanism is proposed. Binding of nematode effectors to the LRR domain of the nematode resistance candidate gene induces conformational changes in the NBS and promotes protein/protein interaction or oligomerization of NBS-LRR proteins. This process could trigger the effector-induced-immune response in Tifguard. Initially, suppression of ABA production and up-regulation of tyrosine, serine-threonine kinases activity may enhance PAMP induced basal immune response. Secondly, activation of ubiquitination may initiate clathrin-coated vesicle formation by labeling nematode effectors with ubiquitin subunits. Regulation of PLD/PA signaling pathways may prepare the necessary modification of cytoskeleton and microtubule architecture to facilitate endocytosis, intracellular trafficking and degradation of nematode effectors. Thirdly, increased mucilage production may hinder nematode feeding site formation.

## Conclusion

This study has provided new mechanistic insight into a major source of root-knot nematode resistance in peanut. Physical mapping of diagnostic SNPs and genetic information from recombinant lines delineated the introgressed region controlling resistance to a 4 Mb region. RNA-seq analysis provided insight into the genetic interaction between host and nematode in the compatible and incompatible context. Finally, the combined RNA-seq analysis and physical mapping led to the identification of a strong candidate gene for nematode resistance.

## Electronic supplementary material

Supplementary Figures

custom script info

custom python script used for analysis

custom python script used for analysis

Table S1

Table S2

Table S3

Table S4

Table S5

Table S6

Table S7

Table S8

Table S9

## References

[1] Abad P (2003). Root-knot nematode parasitism and host response: molecular basis of a sophisticated interaction. Mol Plant Pathol.

[2] Dong WB (2008). Resistance in peanut cultivars and breeding lines to three root-knot nematode species. Plant Dis.

[3] Williamson VM (1996). Nematode pathogenesis and resistance in plants. Plant Cell.

[4] Minton, N. A. & Baujard, P. Nematode parasites of peanut. *Plant parasitic nematodes in subtropical and tropical agriculture*. M. Luc, R. A. Sikora and J. Bridge. Wallingford, UK, CABl, 285–320 (1990).

[5] Milligan SB (1998). The root knot nematode resistance gene *Mi* from tomato is a member of the leucine zipper, nucleotide binding, leucine-rich repeat family of plant genes. Plant Cell.

[6] Claverie M (2011). The *Ma* gene for complete-spectrum resistance to *Meloidogyne* species in Prunus is a TNL with a huge repeated C-terminal post-LRR region. Plant Physiol.

[7] Cook DE (2012). Copy number variation of multiple genes at *Rhg1* mediates nematode resistance in soybean. Science.

[8] Holbrook CC, Noe JP (1992). Resistance to the peanut root-knot nematode (*Meloidogyne arenaria*). In Arachis hypogaea. Peanut Sci.

[9] Holbrook CC, Noe JP (1990). Resistance to *Meloidogyne arenaria* in *Arachis* spp. and the implications on development of resistant peanut cultivars. Peanut Sci.

[10] Nelson SC (1990). Expression of resistance to *Meloidogyne arenaria* in *Arachis batizocoi* and *A*. *cardenasii*. J of Nematol.

[11] Simpson CE (1993). Registration of TxAG-6 and TxAG-7 peanut germplasm lines. Crop Sci.

[12] Simpson CE, Starr JL (2001). Registration of ‘COAN’ peanut. Crop Sci.

[13] Simpson CE (2003). Registration of ‘NemaTAM’ peanut. Crop Sci.

[14] Holbrook CC (2008). Registration of ‘Tifguard’ Peanut. J of Plant Reg.

[15] Nagy ED (2010). Recombination is suppressed in an alien introgression in peanut harboring *Rma*, a dominant root-knot nematode resistance gene. Mol Breed.

[16] Isleib TG (1999). Registration of ‘Gregory’ peanut. Crop Sci.

[17] Chu, Y. *et al*. Identification of rare recombinants leads to tightly linked markers for nematode resistance in peanut. *Peant Sci* accepted (2016).

[18] Tirumalaraju SV (2011). Differential gene expression in roots of nematode-resistant and -susceptible peanut (*Arachis hypogaea*) cultivars in response to early stages of peanut root-knot nematode (*Meloidogyne arenaria*) parasitization. J Plant Physiol.

[19] Norden AJ (1969). Registration of Florunner peanut. Crop Sci.

[20] Jammes F (2005). Genome-wide expression profiling of the host response to root-knot nematode infection in Arabidopsis. Plant J.

[21] Bhattarai KK (2008). Tomato susceptibility to root-knot nematodes requires an intact jasmonic acid signaling pathway. Mol Plant Microbe Interact.

[22] Kandoth PK (2011). The Soybean Rhg1 locus for resistance to the soybean cyst nematode Heterodera glycines regulates the expression of a large number of stress- and defense-related genes in degenerating feeding cells. Plant Physiol.

[23] Ji H (2013). Transcriptional analysis through RNA sequencing of giant cells induced by *Meloidogyne graminicola* in rice roots. J Exp Bot.

[24] Kyndt T (2012). Transcriptional reprogramming by root knot and migratory nematode infection in rice. New Phytol.

[25] Postnikova OA (2015). Transcriptome analysis of resistant and susceptible alfalfa cultivars infected with root-knot nematode Meloidogyne incognita. PLoS One.

[26] Chu Y (2014). A technique to study *Meloidogyne arenaria* resistance in *Agrobacterium rhizogenes*-transformed peanut. Plant Dis.

[27] Haas B (2013). De novo transcript sequence reconstruction from RNA-seq using the Trinity platform for reference generation and analysis. Nat Protoc.

[28] Bertioli, D. J. *et al*. The genome sequences of *Arachis duranensis* and *Arachis ipaensis*, the diploid ancestors of cultivated peanut. *Nat Genet* doi:10.1038/ng.3517 (2016). 10.1038/ng.3517 26901068

[29] Trapnell C (2009). TopHat: discovering splice junctions with RNA-Seq. Bioinformatics.

[30] Li H (2009). 1000 Genome Project Data Processing Subgroup. The Sequence Alignment/Map format and SAMtools. Bioinformatics.

[31] Clevenger JP, Ozias-Akins P (2015). SWEEP: A tool for filtering high-quality SNPs in polyploid crops. G3.

[32] Langmead B (2010). Ultrafast and memory-efficient alignment of short DNA sequences to the human genome. Genome Biol.

[33] Li, B. & Dewey, C. RSEM: accurate transcript quantification from RNA-Seq data with or without a reference genome. *BMC Bioinformatics* **12**, doi: 10.1186/1471-2105-1112-1323 (2011). 10.1186/1471-2105-12-323 PMC3163565 21816040

[34] Love M (2014). Moderated estimation of fold change and dispersion for RNA-seq data with DESeq2. Genome Biol.

[35] Wehrens R, Buydens LMC (2007). Self- and super-organising maps in R: the kohonen package. J Stat Softw.

[36] Andrews, S. FastQC: a quality control tool for high throughput sequence data. Available online at: http://www.bioinformatics.babraham.ac.uk/projects/fastqc (2010).

[37] Li H, Durbin R (2009). Fast and accurate short read alignment with Burrow-wheeler transfmorm. Bioinformatics.

[38] Koenig D (2013). Comparative transcriptomics reveals patterns of selection in domesticated and wild tomato. Proc Natl Acad Sci USA.

[39] Grienenberger E (2010). The interplay of lipid acyl hydrolases in inducible plant defense. Plant Signal Behav.

[40] Church G (2005). A Recessive gene for resistance to *meloidogyne arenaria* in interspecific *Arachis* spp. hybrids. J Nematol.

[41] Hung, C. Y. *et al*. Phosphoinositide-signaling is one component of a robust plant defense response. *Front Plant Sci* **5**, doi: 10.3389/fpls.2014.00267 (2014). 10.3389/fpls.2014.00267 PMC4052902 24966862

[42] Zhang, Q. & Xiao, S. Lipids in salicylic acid-mediated defense in plants: focusing on the roles of phosphatidic acid and phosphatidylinositol 4-phosphate. *Front Plant Sci* **6**, doi: 10.3389/fpls.2015.00387 (2015). 10.3389/fpls.2015.00387 PMC4446532 26074946

[43] Pinosa F (2013). Arabidopsis phospholipase Dδ Is involved in basal defense and nonhost resistance to powdery mildew fungi. Plant Physiol.

[44] Vossen J (2010). Identification of tomato phosphatidylinositol-specific phospholipase-C (PI-PLC) family members and the role of PLC4 and PLC6 in HR and disease resistance. Plant J.

[45] Gheysen G, Mitchum MG (2011). How nematodes manipulate plant development pathways for infection. Curr Opin Plant Biol.

[46] Starr J (1990). Characterization of the resistance to *Meloidogyne arenaria* in an interspecific *Arachis* Spp. hybrid. Peanut Sci.

[47] Grunewald W (2009). Parasitic nematodes modulate PIN-mediated auxin transport to facilitate infection. PLoS Pathog.

[48] Hutangura P (1999). Auxin induction is a trigger for root gall formation caused by root-knot nematodes in white clover and is associated with the activation of the flavonoid pathway. Austr. J Plant Physiol.

[49] Koornneef A, Pieterse CM (2008). Cross talk in defense signaling. Plant Physiol.

[50] Anderson JP (2004). Antagonistic interaction between abscisic acid and jasmonate-ethylene signaling pathways modulates defense gene expression and disease resistance in Arabidopsis. Plant Cell.

[51] Mauch-Mani B, Mauch F (2005). The role of abscisic acid in plant-pathogen interactions. Curr Opin Plant Biol.

[52] Baldridge GD (1998). Alfalfa (*Medicago sativa* L.) resistance to the root-lesion nematode, Pratylenchus penetrans: defense-response gene mRNA and isoflavonoid phytoalexin levels in roots. Plant Mol Biol.

[53] Jones JD (2007). The role of flavonoids produced in response to cyst nematode infection of Arabidopsis thaliana. Nematology.

[54] Berkey R (2012). Sphingolipids and plant defense/disease: the “death” connection and beyond. Front Plant Sci.

[55] Hannun YA, Obeid LM (2002). The Ceramide-centric universe of lipid-mediated cell regulation: stress encounters of the lipid kind. J Biol Chem.

[56] Teixeira MA (2016). Root-knot nematodes induce pattern-triggered immunity in arabidopsis thaliana roots. New Phytol.

[57] Heilmann I (2016). Plant phosphoinositide signaling - dynamics on demand. Biochim Biophys Acta.

[58] Seifi HS (2013). Glutamate metabolism in plant disease and defense: friend or foe?. Mol Plant Microbe Interact.

[59] Blee E (2002). Impact of phyto-oxylipins in plant defense. Trends Plant Sci.

[60] Goellner M (2001). Endo-beta-1,4-glucanase expression in compatible plant-nematode interactions. Plant Cell.

[61] de Almeida Engler J (2004). Dynamic cytoskeleton rearrangements in giant cells and syncytia of nematode-infected roots. Plant J..

[62] Lerouxel O (2006). Biosynthesis of plant cell wall polysaccharides - a complex process. Curr Opin Plant Biol..

[63] Mitchum MG (2004). The promoter of the Arabidopsis thaliana Cel1 endo-1,4-b-glucanase gene is differentially expressed in plant feeding cells induced by rootknot and cyst nematodes. Mol Plant Pathol..

[64] Sobczak, M. *et al*. Cell wall modification induced by nematodes. *Genomics and molecular genetics of plant-nematode interactions*. Springer Sciences Business Media, 395–422 (2011).

[65] Clement M (2009). Actin-Depolymerizing Factor2-mediated actin dynamics are essential for root-knot nematode infection of Arabidopsis. Plant Cell.

[66] Nahar K (2012). Abscisic acid interacts antagonistically with classical defense pathways in rice-migratory nematode interaction. New Phytol.

[67] Chen X (2011). Clathrin-mediated endocytosis: the gateway into plant cells. Curr Opin Plant Biol.

[68] Frei dit Frey N, Robatzek S (2009). Trafficking vesicles: pro or contra pathogens?. Curr Opin Plant Biol.

[69] Robatzek S (2006). Ligand-induced endocytosis of the pattern recognition receptor FLS2 in Arabidopsis. Genes Dev.

[70] Ichimura K (2002). Mitogen-activated protein kinase cascades in plants: a new nomenclature. Trends Plant Sci..

[71] Afzal AJ (2008). Plant receptor-like serine threonine kinases: roles in signaling and plant defense. Mol Plant Microbe Interact.

[72] Hawes MC (2000). The role of root border cells in plant defense. Trends Plant Sci.

[73] Hawes MC, Pueppke SG (1987). Correlation between binding of *Agrobacterium tumefaciens* by root cap cells and susceptibility of plants to crown gall. Plant cell Re.

[74] Perez-de-Luque A (2006). Mucilage production during the incompatible interaction between Orobanche crenata and Vicia sativa. J Exp Bot.

[75] Bargmann BO, Munnik T (2006). The role of phospholipase D in plant stress responses. Curr Opin Plant Biol.

[76] Zhao J (2015). Phospholipase D and phosphatidic acid in plant defence response: from protein-protein and lipid-protein interactions to hormone signalling. J Exp Bot.

[77] Kaplan B (2007). Cyclic nucleotide-gated channels in plants. FEBS Lett.

[78] Kochert G (1996). RFLP and cytogenetic evidence on the origin and evolution of allotetraploid domesticated peanut, *Arachis hypogaea* (Leguminosae). Am J Botany.

[79] Chisholm ST (2006). Host-microbe interactions: shaping the evolution of the plant immune response. Cell.

[80] Tameling WI (2002). The tomato R gene products I-2 and *MI*-1 are functional ATP binding proteins with ATPase activity. Plant Cell.

[81] Feys BJ, Parker JE (2000). Interplay of signaling pathways in plant disease resistance. Trends Genet.

[82] Paal J (2004). Molecular cloning of the potato Gro1-4 gene conferring resistance to pathotype Ro1 of the root nematode Globodera rostochiensis, based on a candidate gene approach. Plant J.

[83] Jablonska B (2007). The Mi-9 gene from Solanum arcanum conferring heat-stable resistance to root-knot nematodes is a homolog of Mi-1. Plant Physiol.

[84] Ernst K (2002). The broad-spectrum potato cyst nematode resistance gene *(Hero*) from tomato is the only member of a large gene family of NBS-LRR genes with an unusual amino acid repeat in the LRR region. Plant J.

[85] DeYoung BJ, Innes RW (2006). Plant NBS-LRR proteins in pathogen sensing and host defense. Nat Immunol.

