# Supplementary material for: Gene expression profiling describes the genetic regulation of Meloidogyne arenaria resistance in Arachis hypogaea and reveals a candidate gene for resistance
Source: Sci Rep. 2017 May 2;7:1317. doi: 10.1038/s41598-017-00971-6 (PMC5430994; doi:10.1038/s41598-017-00971-6)
Supplement: Supplementary file 1 — Supplementary Figures [file 41598_2017_971_MOESM1_ESM.docx]

RNA sequencing describes the genetic regulation of *Meloidogyne arenaria* resistance in *Arachis hypogaea* and reveals a candidate gene for resistance

Josh Clevenger, Ye Chu, Larissa Guimaraes, Thiago Maia, David Bertioli, Soraya Leal-Bertioli, Patricia Timper, C. Corley Holbrook, and Peggy Ozias-Akins

**Supplementary Figures**

**
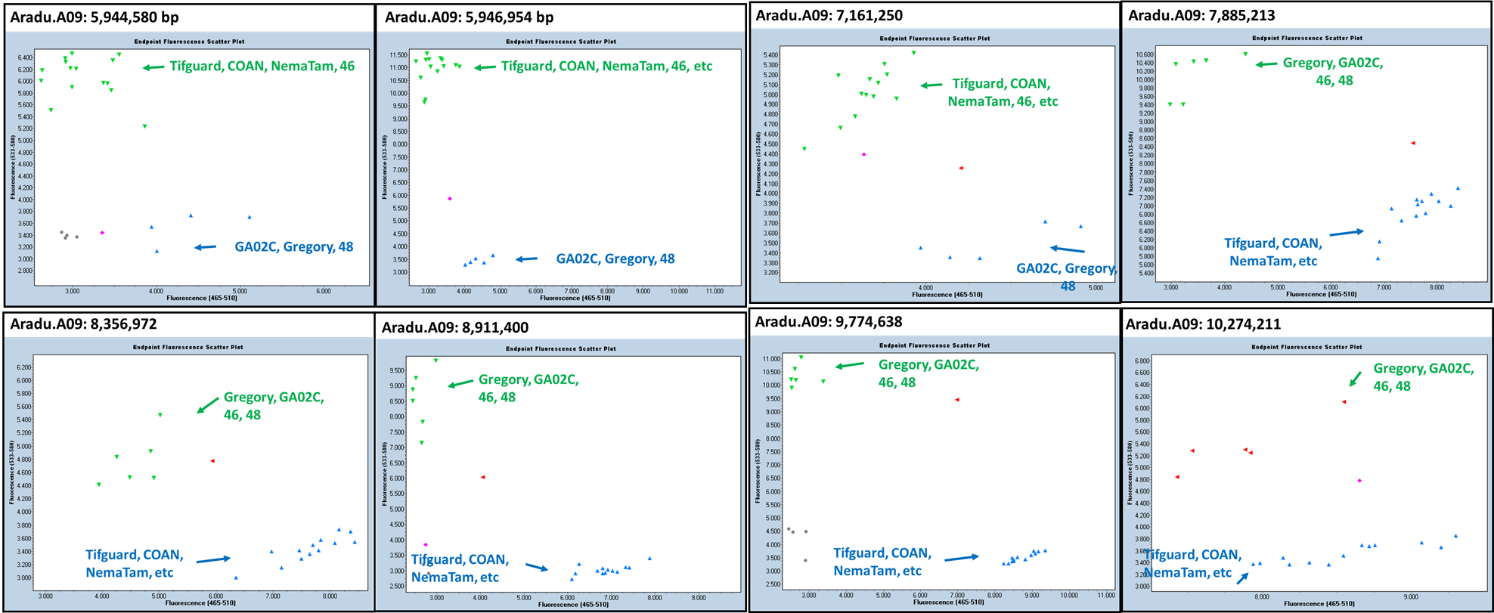
**

**Figure S1: Eight KASP markers showing confirmation of the SNP mapping covering the small introgression in Line 46.** The position on pseudomolecule A09 (*A. duranensis*) for each SNP is in the upper left corner. For each marker Tifguard, COAN, and NemaTam are shown for controls of the resistant allele and GA02C and Gregory are shown for the susceptible allele. Line 46 shares the allele of Tifguard for markers upstream of 7,161,250 and shares alleles with Gregory downstream of 7,885,213, corroborating the *in sillico* SNP mapping.

**
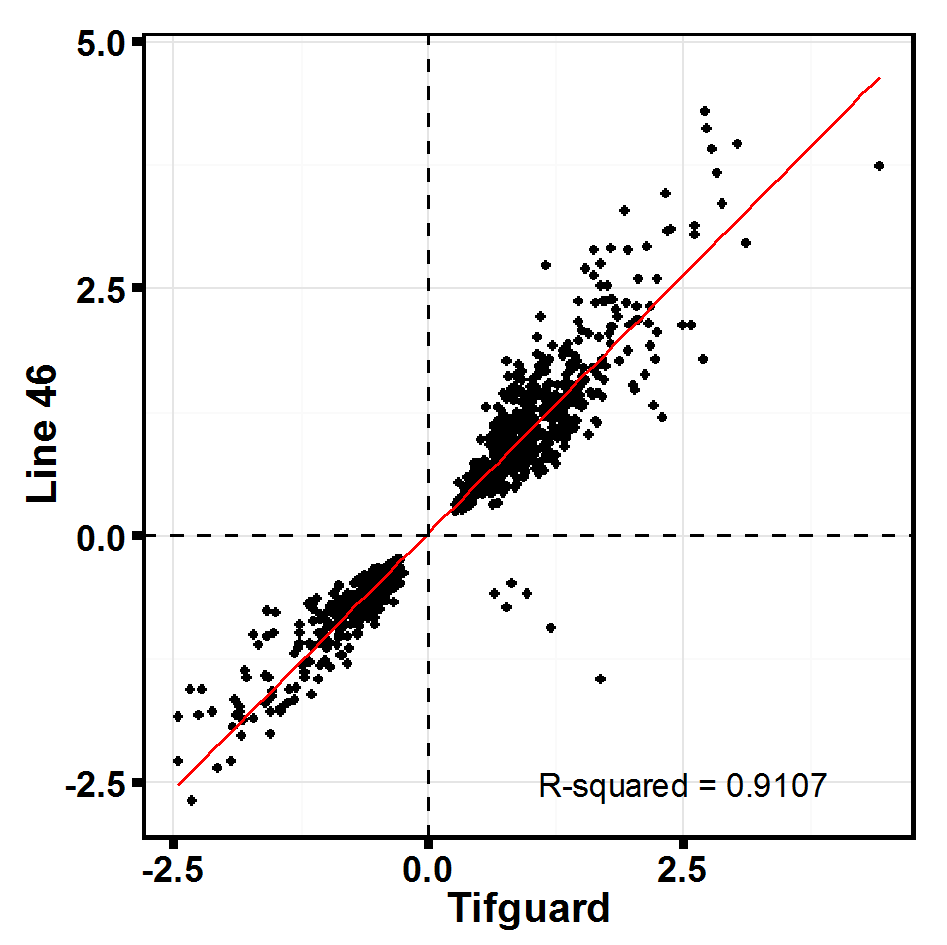
**

**Figure S2:** log transformed fold change estimates of genes significantly affected by genotype x treatment in the comparison of Tifguard vs Gregory (x axis) and line 46 and Gregory (y axis) are highly correlated (R-square = 0.91)**.**

**
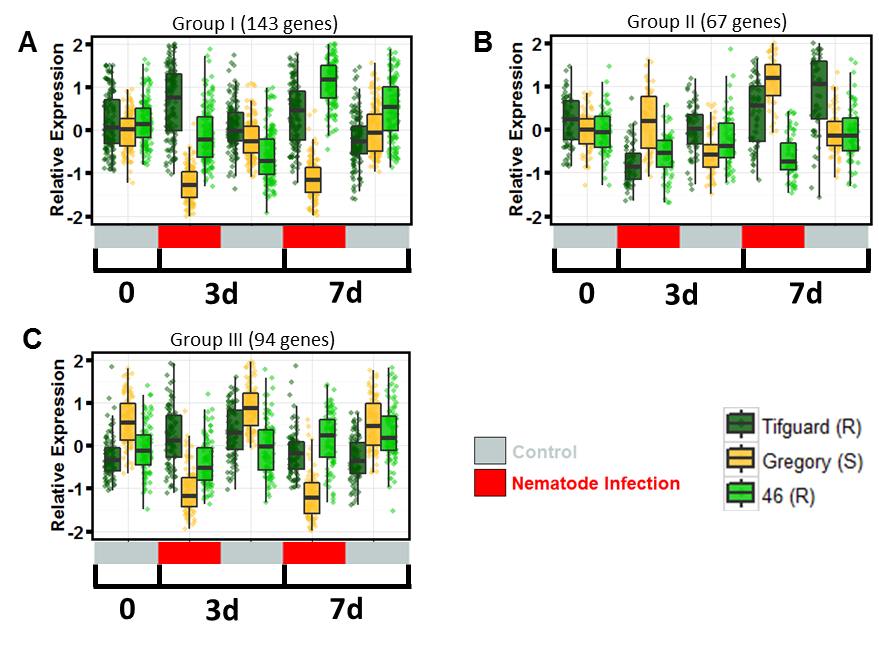
**

**Figure S3: Co-expression networks of differentially expressed genes between line 46 and Gregory affected by genotype x treatment.**  Tifguard is shown for comparison purposes. A) Group I B) Group II C) Group III

**
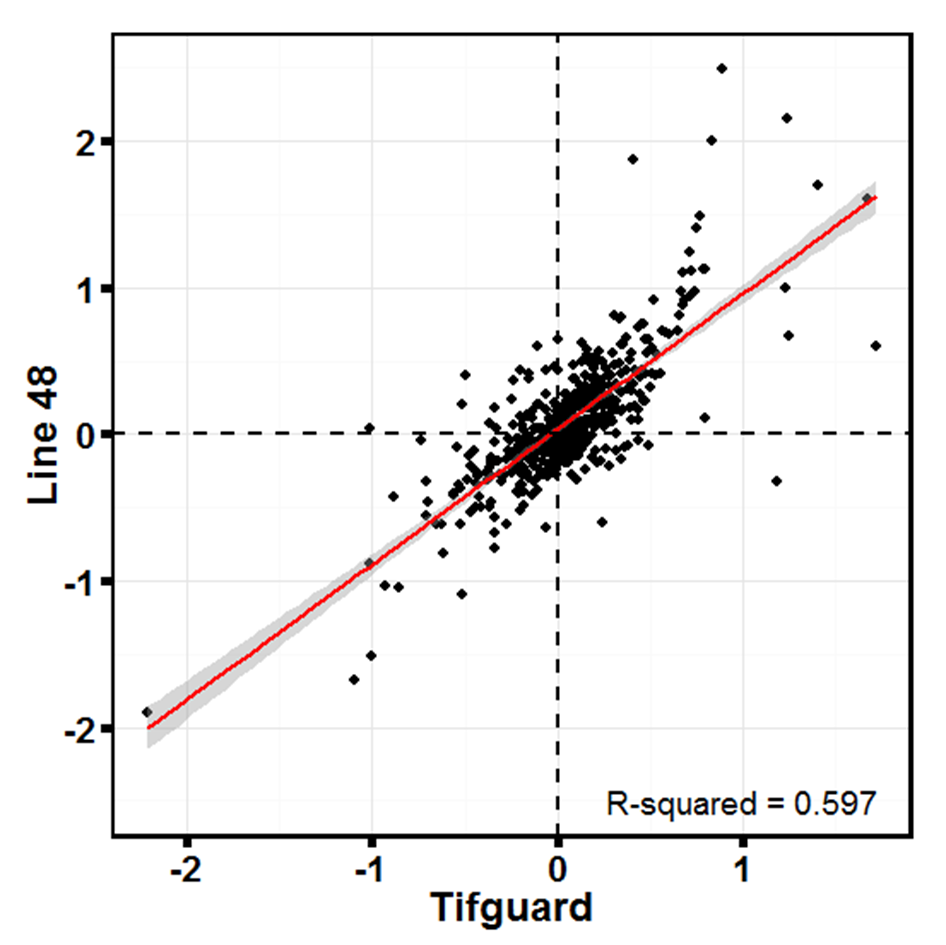
**

**Figure S4:** log transformed fold change estimates of genes significantly affected by genotype in the comparison of Tifguard vs Gregory (x axis) and line 48 and Gregory (y axis) are significantly correlated (R-square = 0.591; p < 0.001)**.**

**
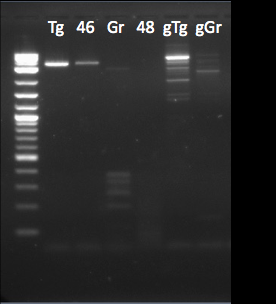
**

**Figure S5:** RT-PCR targeting the candidate resistance gene in Tifguard. From left to right, lane 1 – Tifguard cDNA, lane 2 – line 46 cDNA, lane 3 – Gregory cDNA, lane 4 – line 48 cDNA, lane 5 – Tifguard genomic DNA, and lane 6 – Gregory genomic DNA.

**
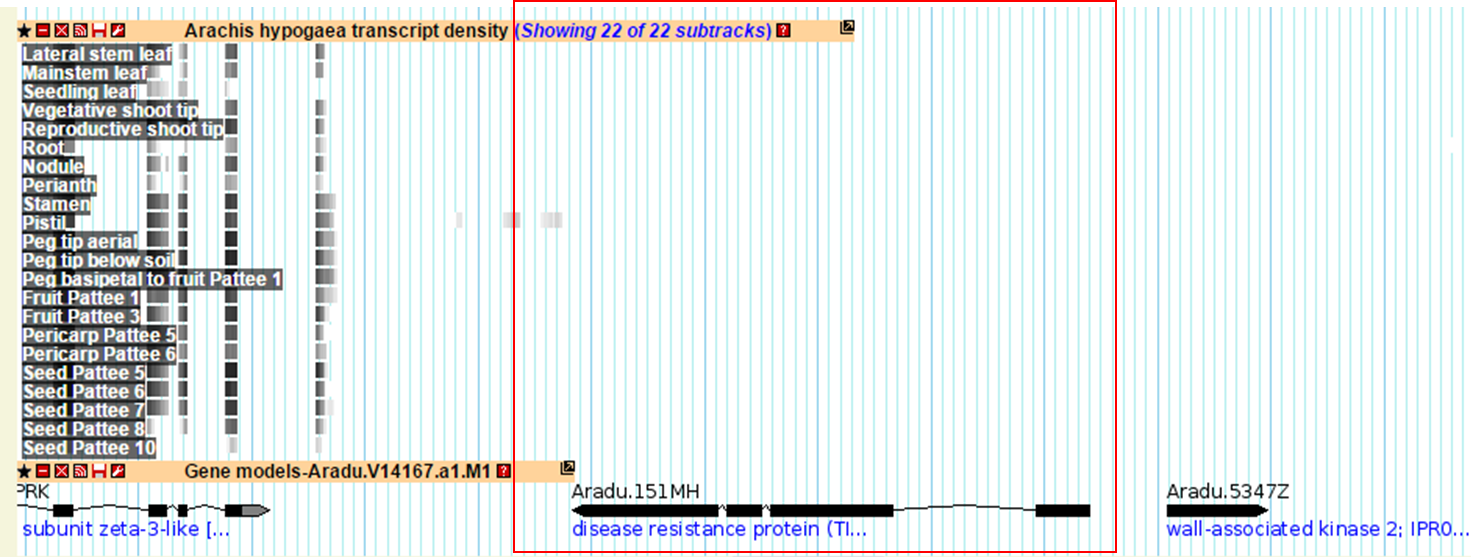
**

**Figure S6:** Screen shot of peanutbase.org *A. duranensis* genome browser with expression tracks for the region that the candidate gene maps to. A NBS-TIR R gene is predicted in the region, but is also not expressed at all in *A. hypogaea* C.V. Tifrunner.


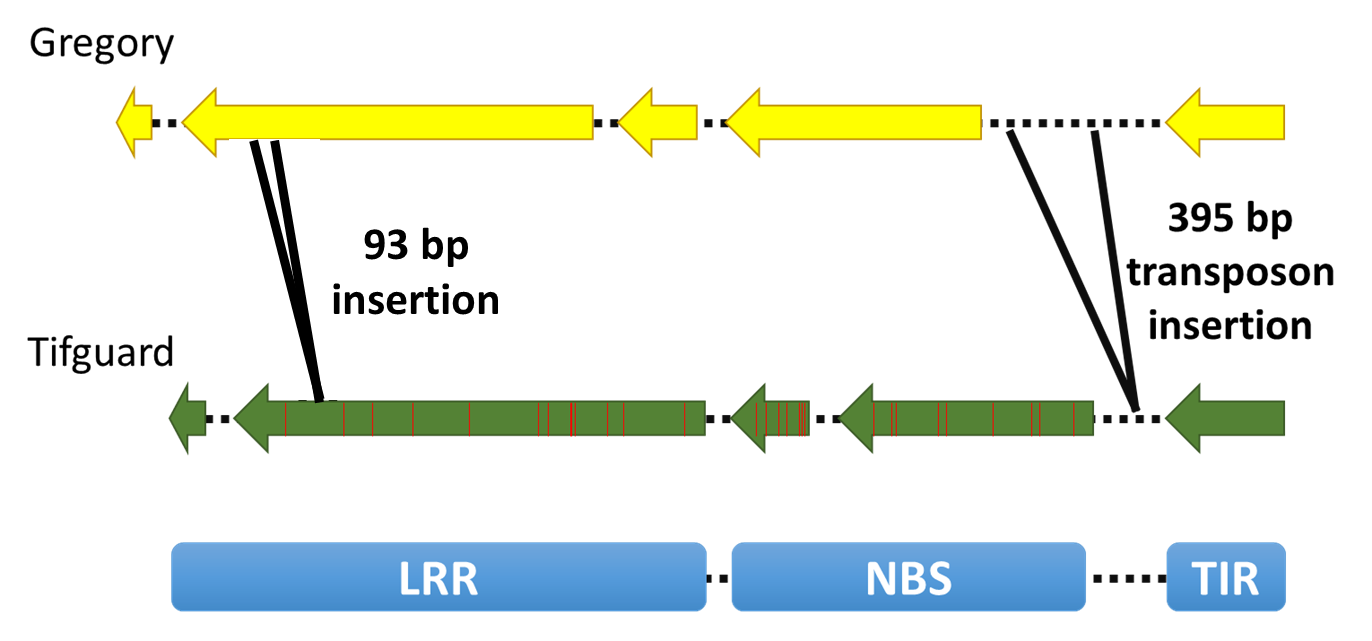


**94 bp**

**397 bp**

**Figure S7:** Illustration of structural variation in candidate region of Tifguard (*A. cardenasii* lineage) and Gregory (*A. duranensis* lineage). SNPs are shown as red lines in Tifguard exon models. Predicted regions (blue boxes) corresponding to main regions of NBS-TIR R gene are predicted using FGENESH (www.softberry.com).





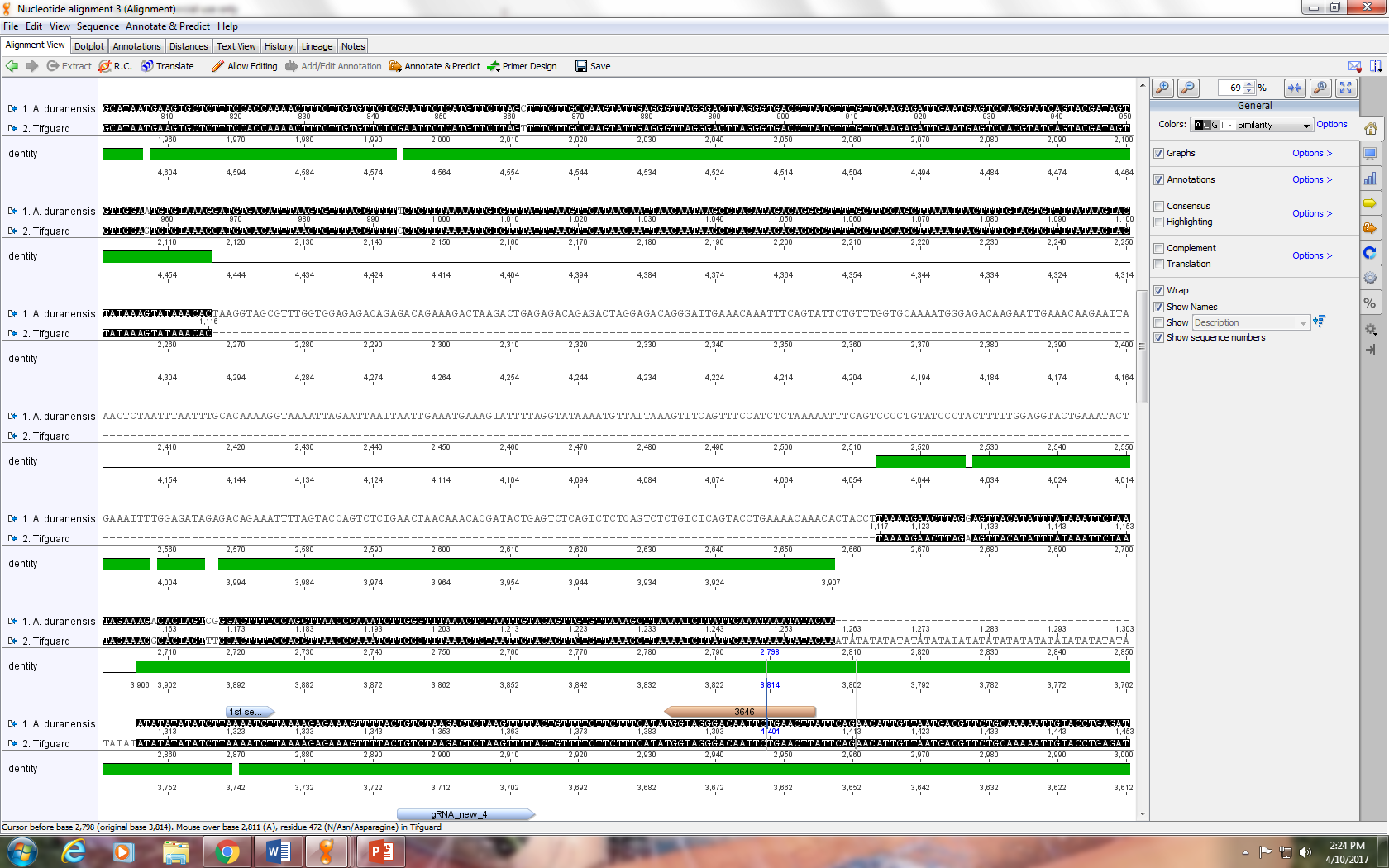


397 bp insertion in *A. duranensis* in intron 1


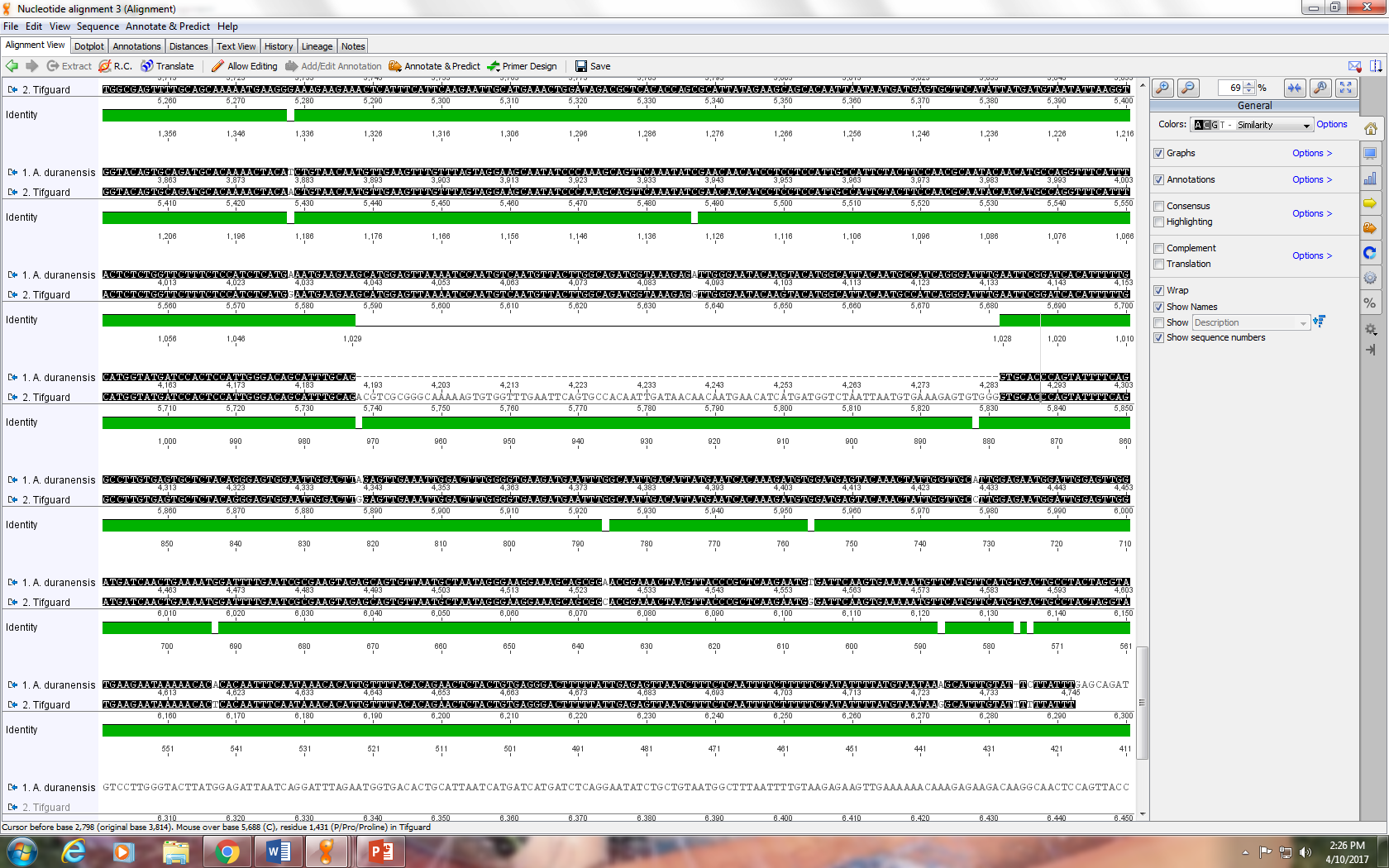


94 bp insertion in Tifguard in exon 4

**Figure S8:** Genomic DNA from Tifguard (R), RIL46 (R), Georgia Green (nematode susceptible peanut cultivar), *A. duranensis* V14167 (A genome progenitor), *A. ipaensis* KG30067 (B genomic progenitor), *A. cardenasii* (A genome nematode resistance donor) were amplified with Sense 5’-TGTAAAACGACGGCCAGTTGTCTTCCTCCGGCAGAAAG-3’; antisense 5’-TGAAAGTTCAATCCCTTGAGTTCA-3’ covering exon 1- intron 1 – exon2 of the candidate gene. Same size fragment was amplified from Tifguard, RIL46 and *A. cardenasii* which supports the *A. cardenasii* origin of the candidate gene. An approximately 400 bp larger fragment was amplified in *A. duranensis*. Further Sanger sequencing analysis identified this 397 bp insertion is located in intron 1. The absence of amplicon in *A. ipaensis* suggests that there is no similar sequence to the candidate gene in B genome. The absence of amplicon in Georgia Green suggests major modification of this A genome region such as gene deletion, conversion etc. may occur after the polyploidization event that gave rise to cultivated peanut. Alignments below show insertion in intron 1 in *A. duranensis* (above) using Sanger sequencing and the insertion in exon 4 in *A. cardenasii*/Tifguard (below).


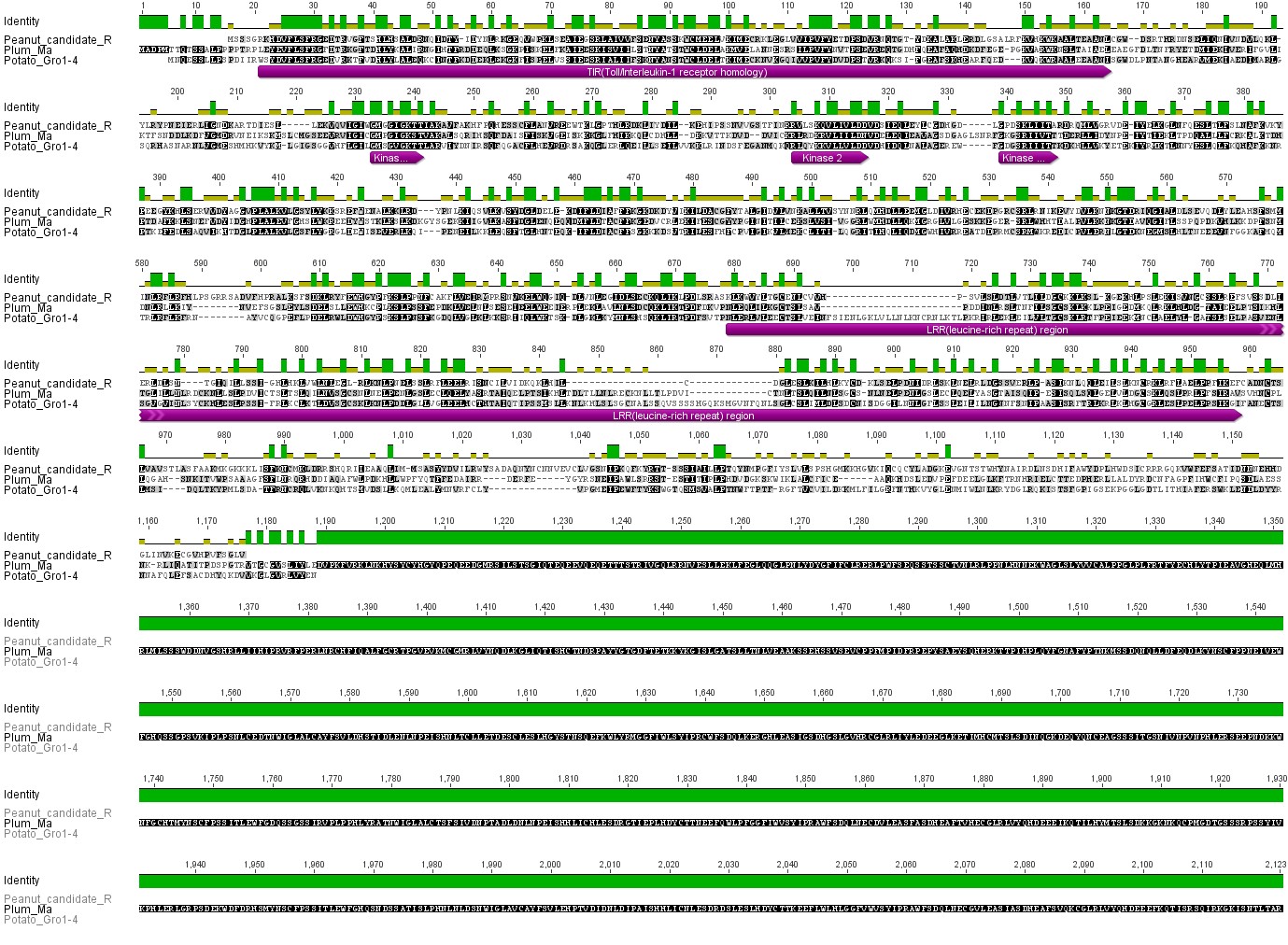


Figure S9. Alignment of candidate R gene protein sequence with previously identified nematode resistance genes Gro 1-4 and Ma TNL 1 in potato and plum respectively.
