# Supplementary material for: Gene expression profiling describes the genetic regulation of Meloidogyne arenaria resistance in Arachis hypogaea and reveals a candidate gene for resistance
Source: Sci Rep. 2017 May 2;7:1317. doi: 10.1038/s41598-017-00971-6 (PMC5430994; doi:10.1038/s41598-017-00971-6)
Supplement: Supplementary file 2 — custom script info [file 41598_2017_971_MOESM2_ESM.pdf]

##Scripts readme

##First filter vcf files of species of interest against reference of interest and genotype of interest with as many control genotypes as available using FilterIntro.py.

##Second run MapIntro.py to select those SNPs that are diagnostic in the species of interest and are unique in the genotype of interest relative to the control genotypes
