# Supplementary material for: Gene expression profiling describes the genetic regulation of Meloidogyne arenaria resistance in Arachis hypogaea and reveals a candidate gene for resistance
Source: Sci Rep. 2017 May 2;7:1317. doi: 10.1038/s41598-017-00971-6 (PMC5430994; doi:10.1038/s41598-017-00971-6)
Supplement: Supplementary file 3 — custom python script used for analysis [file 41598_2017_971_MOESM3_ESM.pdf]

```
import sys

##Function to genotype the vcf SNP calls from the PL values
def get_genotype(line, number):
     genotype = []
     for x in range(number):

          prob = line[x].split(',')
          if len(prob) > 3:
               return 0
          if int(prob[0]) == 0 and int(prob[1]) > 0 and int(prob[2]) > 0:
               genotype.append(1)

          if int(prob[0]) > 0 and int(prob[1]) == 0 and int(prob[2]) > 0:
               genotype.append(2)

          if int(prob[0]) > 0 and int(prob[1]) > 0 and int(prob[2]) == 0:
               genotype.append(3)

          if int(prob[0]) == 0 and int(prob[1]) >= 0 and int(prob[2]) ==
0:
               genotype.append('N')

          if int(prob[0]) > 0 and int(prob[1]) > 0 and int(prob[2]) > 0:
               genotype.append('N')
     return genotype

##vcf file with introgressed genotype and control genotypes
targetfile = open(sys.argv[1], "r")

for line in targetfile:

     line = line.strip()
     hold = line.split()
     if hold[0].startswith('#'):
          continue

     result = get_genotype(hold[9:13],4)


     ##select those SNPS that are present in introgressed genotype and
not in control genotypes
     if result:
          if len(result) < 4:
               continue
          if result[0] == 3 and result[1] == 1 and result[2] == 1 and
result[3] == 1:
               print(line)

targetfile.close()
```
