# Supplementary material for: Gene expression profiling describes the genetic regulation of Meloidogyne arenaria resistance in Arachis hypogaea and reveals a candidate gene for resistance
Source: Sci Rep. 2017 May 2;7:1317. doi: 10.1038/s41598-017-00971-6 (PMC5430994; doi:10.1038/s41598-017-00971-6)
Supplement: Supplementary file 4 — custom python script used for analysis [file 41598_2017_971_MOESM4_ESM.pdf]

```
import sys

##species specific SNPs filtered file
wildfile = open(sys.argv[1], "r")

##target genotype filtered file
targetfile = open(sys.argv[2], "r")

chr = ''
pos = ''
ref = ''
alt = ''

wild = {}

##put species-specific in a hash
for line in wildfile:

     line=line.strip()
     line=line.split()

     if line[0].startswith('#'):
          continue

     wild[line[0]+":"+line[1]+":"+line[3]+":"+line[4]] = 1

##select overlap as introgressed regions
for x in targetfile:

     x=x.strip()
     hold=x.split()

     chr=hold[0]
     pos=hold[1]
     ref=hold[3]
     alt=hold[4]

     if (chr+":"+pos+":"+ref+":"+alt) in wild:

          print(x)

wildfile.close()
targetfile.close()
```
